# Natural Variation of the Amino-Terminal Glutamine-Rich Domain in *Drosophila* Argonaute2 Is Not Associated with Developmental Defects

**DOI:** 10.1371/journal.pone.0015264

**Published:** 2010-12-17

**Authors:** Daniel Hain, Brian R. Bettencourt, Katsutomo Okamura, Tibor Csorba, Wibke Meyer, Zhigang Jin, Jason Biggerstaff, Haruhiko Siomi, Gyorgy Hutvagner, Eric C. Lai, Michael Welte, H.-Arno J. Müller

**Affiliations:** 1 Division of Cell and Developmental Biology, College of Life Sciences, University of Dundee, Dundee, United Kingdom; 2 Alnylam Pharmaceuticals, Cambridge, Massachusetts, United States of America; 3 Sloan-Kettering Institute, Department of Developmental Biology, New York, New York, United States of America; 4 Wellcome Trust Centre for Gene Regulation and Expression, College of Life Sciences, University of Dundee, Dundee, United Kingdom; 5 Institut für Genetik, Heinrich Heine Universität, Düsseldorf, Germany; 6 Boston University, Boston, Massachusetts, United States of America; 7 Department of Molecular Biology, Keio University School of Medicine, Tokyo, Japan; 8 Department of Biology, University of Rochester, Rochester, New York, United States of America; University of Texas MD Anderson Cancer Center, United States of America

## Abstract

The *Drosophila argonaute2* (*ago2*) gene plays a major role in siRNA mediated RNA silencing pathways. Unlike mammalian Argonaute proteins, the *Drosophila* protein has an unusual amino-terminal domain made up largely of multiple copies of glutamine-rich repeats (GRRs). We report here that the *ago2* locus produces an alternative transcript that encodes a putative short isoform without this amino-terminal domain. Several *ago2* mutations previously reported to be null alleles only abolish expression of the long, GRR-containing isoform. Analysis of *drop out* (*dop*) mutations had previously suggested that variations in GRR copy number result in defects in RNAi and embryonic development. However, we find that *dop* mutations genetically complement transcript-null alleles of *ago2* and that *ago2* alleles with variant GRR copy numbers support normal development. In addition, we show that the assembly of the central RNAi machinery, the RISC (RNA induced silencing complex), is unimpaired in embryos when GRR copy number is altered. In fact, we find that GRR copy number is highly variable in natural *D. melanogaster* populations as well as in laboratory strains. Finally, while many other insects share an extensive, glutamine-rich Ago2 amino-terminal domain, its primary sequence varies drastically between species. Our data indicate that GRR variation does not modulate an essential function of Ago2 and that the amino-terminal domain of Ago2 is subject to rapid evolution.

## Introduction

Argonaute proteins play key roles in diverse mechanisms of gene regulation from plants to fungi and animals, including humans. The Argonaute protein family is characterized by a stereotyped domain structure and has been divided into the Argonaute subfamily (related to *Arabidopsis thaliana* Argonaute) and the PIWI subfamily (related to *D. melanogaster* PIWI) [1], [2]. Organisms may contain a large number of different Argonaute encoding genes, currently with a maximum of 26 family members identified in *C. elegans* [3]. The presence of multiple, differentially expressed family members in a single species indicates diverse biological functions of Argonaute proteins.

A common feature of all Argonaute proteins is that they bind small RNAs and mediate silencing of target transcripts by translational inhibition or RNA degradation [4]. Argonaute subfamily proteins interact with siRNAs and/or miRNAs and in animals exert diverse functions during development of various somatic lineages. These functions range from selective degradation of unwanted maternal mRNAs in early development to the regulation of transcripts important for cell fate decisions during neuronal and muscle development as well as degradation of viral RNA in adult organisms [5]. An important function for the RNAi pathway was also demonstrated in innate immune response against viruses in animals and plants [6], [7], [8], [9].

*Drosophila* has proved to be an excellent model system for research on Argonaute protein function [10], [11], [12]. The *Drosophila melanogaster* genome encodes five Argonaute family members: Piwi subfamily members Aubergine (Aub), Piwi and Ago3 and the Argonaute subfamily members Ago1 and Ago2. Ago1 is essential for miRNA function, while Ago2 predominantly acts in the siRNA pathway [11], [13], [14]. Ago2 is the carrier of both exogenous and endogenous siRNAs, and functions in virus defense, restriction of transposable elements, and cleavage of mRNA targets [15]. Ago1 and Ago2 also have overlapping functions, *e.g.* in the control of segment polarity during embryogenesis [16].

*Drosophila* Ago2 is unique among the Argonaute subfamily: Its sequence is highly divergent and clearly distinct from other metazoan Argonautes (including *Drosophila* Ago1 and the four human Argonautes Ago1, Ago2, Ago3 and Ago4) or even Argonautes from fungi or plants [17]. It is a modular protein with the canonical PAZ and PIWI domains in the carboxy-terminal half and an amino-terminal half that is highly enriched in glutamine residues [16]. The carboxy-terminal region (820 amino acids) is broadly conserved in Argonaute family members from plants to fission yeast and vertebrates and has well-characterized biochemical functions, such as binding and cleavage of RNA [1]. In contrast, the amino-terminal portion (in the following referred to as the amino-terminal domain or NTD) is not well characterized. While the corresponding region is typically very short in most other family members (e.g. 24 aa in human Ago2), the Ago2 NTD in *D. melanogaster* is 397 amino acids long and encompasses long stretches of low sequence complexity (40% of all residues are glutamine). The majority of the NTD consists of two types of imperfect glutamine-rich repeats (GRR), called GRR1 (6 amino acid imperfect repeat) and GRR2 (23 amino acid imperfect repeat) ([16]; see below). Previous analysis of the *drop out* (*dop*) mutation suggested that altered GRR copy number disrupts Ago2 function and leads to maternal-effect lethality [16].

In the present paper, we demonstrate that *ago2* is a complex locus that produces several distinct transcripts. The protein isoforms encoded by these transcripts have identical C-terminal regions that include the PAZ and Piwi domains. But while two long isoforms contain the unusual NTD, a previously uncharacterized short isoform lacks both types of amino-terminal GRRs, reminiscent of mammalian Argonaute proteins. We find that two *ago2* mutant alleles widely employed as null alleles still express the short isoform. Using true null alleles, we show that the *dop* mutation is not allelic to *ago2* and that GRR variation does not cause striking defects in embryonic development. In fact, we find extensive GRR repeat-number variation among *D. melanogaster* strains isolated from natural and laboratory populations as well as tremendous variability of the Ago2 NTD between insect species. These data suggest that this domain is subject to rapid evolution and has a non-essential, possibly modulatory function.

## Results and Discussion

### The *ago2* locus generates transcripts encoding putative long and short isoforms

**Figure 1:**
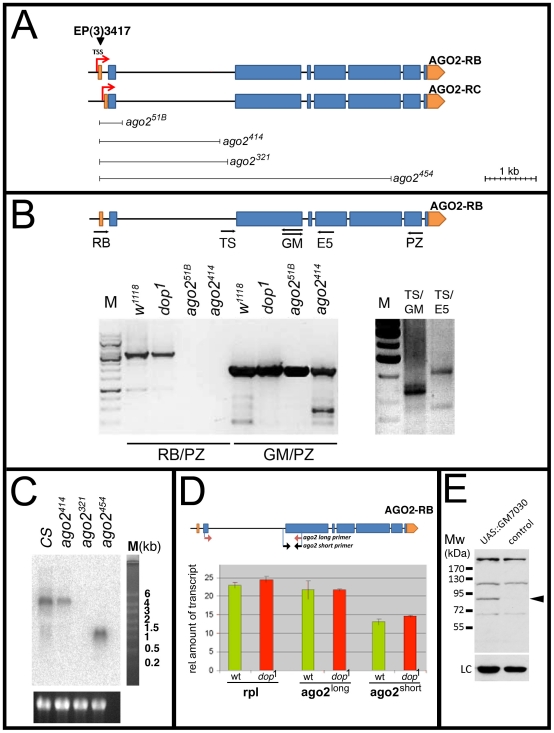
The *ago2^51B^* and *ago2^414^* alleles produce an alternative transcript encoding a putative short form of the protein. (**A**) *ago2* genomic organization indicating 8 exons of the two alternatively spliced gene products Ago2-RB and Ago2-RC (UTRs are indicated in orange; CDS is indicated in blue). Also shown is the extent of the chromosomal deletions generated by imprecise excision of the *EP(3)3417* P-element insertion, located 12 bp downstream of the transcriptional start site (TSS) for Ago2-RB. The *ago2^414^* allele creates a deletion of 2356 bp and *ago2^321^* creates a deletion of 2514 bp. (**B**) Cartoon of genomic organization of Ago2-RB and the positions of the PCR primers (RB, TS, GM (forward and reverse, respectively), E5, and PZ). RT-PCR experiments using primer pairs as indicated; all PCR products were subjected to DNA sequencing. The RB/PZ primer pair amplifies a product of 3488 bp indicative of a transcript encoding for the long isoform; this product is only detected in *w^1118^* and *dop^1^*, but not in *ago2^51B^* and *ago2^414^*. GM/PZ amplifies a 2200 bp product, which is detected in all genotypes. The TS primer was designed to bind within the second intron. The TS/GM or TS/E5 primer pairs revealed products of the predicted sizes and sequences of an alternative transcript encoding for the short isoform (*w^1118^* samples). All template polyA^+^ RNA was prepared from ovaries. (**C**) 10 µg total RNA from adult flies was subjected to Northern blotting. The *ago2^414^* allele produces detectable *ago2* transcripts whereas no transcript was detected in *ago2^321^* homozygous flies. Note that the probe was designed against the downstream of *ago2^454^* deletion and a putative truncated mRNA was detected in *ago2^454^* homozygous flies. (**D**) Quantitative RT PCR comparing expression of transcripts encoding Ago2^long^ and Ago2^short^ from total RNA of 3–5 hrs embryos. Primer pairs were designed to specifically amplify PCR products of transcripts encoding for the short and long isoform, respectively. (**E**) *UAS::GM07030-HA* was expressed in embryos using maternal *α4-tubulin64C::Gal4* (mat67;mat15) and proteins were immunoblotted using anti-HA antibody. *w* ^1118^ embryos were used as a control. The arrowhead indicates one specific protein band at around 85 kDa indicative of HA-tagged Ago2^short^ (LC - loading control).

Gene annotation predicts that the *ago2* locus gives rise to the two transcripts AGO2-RB and AGO2-RC (FlyBase, 6_2010). They are largely identical, but differ at their very 5′ ends, due to alternative splicing and the use of distinct transcription start sites (Fig. 1A). As a result, the two protein isoforms encoded by these transcripts, AGO2-PB and AGO2-PC, differ only by tiny unique stretches (6 or 9 amino acids) at their amino termini and have a 1208 amino acid region in common.

To functionally characterize Ago2, many studies employ the two putative null alleles *ago2^51B^* and *ago2^414^*. These alleles represent two independently derived deletions that eliminate the first two annotated AGO2-RB exons and part of the third intron (Fig. 1A; [11], [18]). As they delete the annotated translational start sites for both AGO2-RB and AGO2-RC, these alleles are expected to abolish all *ago2* expression [11], [18].

To test this prediction directly, we performed RT-PCR analysis with various primers across the *ago2* locus (Fig. 1B). Experiments that involved a primer specific for exon 1 (primer RB) failed to detect any *ago2* transcripts in these alleles (Fig. 1B), confirming the absence of AGO2-RB and AGO2-RC. However, when we employed primers from exons 3 through 7, we detected amplification products in these supposed null alleles. These products are not due to contamination with genomic DNA templates as their size was the same as in the wild type and sequencing confirmed that they represent correctly spliced transcripts. Furthermore, Northern analysis of total RNA of adult flies confirmed the presence of *ago2* transcript in *ago^414^* (Fig. 1C). These results indicate the presence of previously unannotated transcripts in *ago2^51B^* and *ago2^414^*.

These transcripts could also be detected with primers upstream of the third exon splice site, a region annotated as intronic for AGO2-RB and AGO2-RC (Fig. 1B, primer TS). Apparently, the alternate transcripts originate 5′ to exon 3. This property makes it possible to design isoform-specific primers to examine the expression of the alternative transcript and of AGO2-RB/AGO2-RC separately. Using these primers, we found that wild-type flies express both types of transcripts. Quantitative RT-PCR analysis on samples from adult flies indicates that the shorter transcript is expressed approximately 30% less than AGO2-RB/AGO2-RC (Fig. 1D).

These alternative transcripts are predicted to encode a shorter protein isoform of Ago2, in the following referred to as Ago2^short^. Since we have not been able to verify protein production from this transcript in vivo, we tested whether a cDNA encoding Ago2^short^ can in principle produce a protein of the expected size. The Berkeley *Drosophila* genome project (BDGP) had isolated an EST clone, *GM07030*, which is predicted to encode Ago2^short^. We generated a transgene, in which HA-tagged *GM07030* is driven under the control of UAS elements. Uniform expression of this transgene in the embryo results in a protein product of approximately 85 kDa, consistent with the predicted molecular weight of Ago2^short^ (Fig. 1E).

A truncated Ago2 protein lacking GRRs was recently shown to support full biochemical reconstitution of RISC indicating that the N-terminal region does not contain domains that are essential for core RNAi [19]. In the lack of an NTD, Ago2^short^ is reminiscent of the mammalian Argonautes; indeed, BLAST comparisons reveal significant similarity between Ago2^short^ and human Ago2 throughout essentially the entire length of the proteins (not shown). We conclude that the *ago2* locus has the potential to encode at least three protein isoforms, only two of which encompass the unusual NTD that sets *D. melanogaster* Ago2 apart from other family members.

### Identification of true null alleles of *ago2*

The available putative null alleles of *ago2* remove 5′ exons with limited coding potential [11], [18]. The existence of an alternative isoform implies that *ago2^51B^* and *ago2^414^* may retain some Ago2 function. Indeed, although Northern analysis of embryos had suggested that *ago2^414^* is transcript null [11], we observed substantial amounts of nearly full length transcript in adult flies (Fig. 1C).

We therefore examined additional excision events derived from *EP(3)3417* to identify bona fide Ago2 null alleles. This screen identified two alleles that were transcript null in the adult, *ago2^321^* and *ago2^454^* (Fig. 1C). These alleles contain the same 5′ breakpoint located 34 bp downstream of the transcriptional start site of AGO2-RB, but have different 3′ breakpoints (Fig. 1A). The deletion in *ago2^321^* removes 2514 bp and is very similar to *ago2^414^*, but retains a section of the original EP element; this insertion may contribute to why it is transcript null. *ago2^454^* carries a deletion of 5750 bp and accumulates a truncated transcript consistent with this deletion. This deletion removes the PAZ domain and much of the PIWI domain, and is therefore unquestionably a null allele.

Homozygotes of *ago2^321^* and *ago2^454^* stocks were viable, but unlike *ago2^51B^* and *ago2^414^*, they exhibited low fertility in both males and females. 55% of embryos obtained from *ago2^321^* homozygous mothers did not hatch (n = 221), while *ago2^454^* homozygous females did not lay any eggs. 50% ovaries from *ago2^321^* and 90% ovaries from *ago2^454^* homozygotes are rudimentary. Surprisingly, none of the phenotypes seen in *ago2^321^* or *ago2^454^* homozygotes except male sterility were seen in *ago2^321^/ago2^454^* trans-heterozygotes. Moreover, *ago2^321^/Df* or *ago2^454^/Df* hemizygotes (using the molecularly defined deficiencies *Df(3L)ED218* and *Df(3L)BSC5580*) displayed none of the above-mentioned phenotypes. Therefore, the fertility phenotypes observed in *ago2^321^* and *ago2^454^* homozygotes are likely attributable to background mutations. While a detailed analysis of these novel *ago2* null alleles is beyond the scope of this paper, an exciting prospect for the future is to determine whether these alleles corroborate or extend other phenotypes that were previously reported for *ago2^414^* or *ago2^51B^* alleles. Such phenotypes include defective heterochromatin formation [20], defective neuromuscular junction and egg chamber development [21], and defects in embryonic syncytial divisions and pole cell formation [22]. The partial penetrance of these defects in *ago2^414^* and *ago2^51B^* homozygotes might result from residual Ago2 function due to Ago2^short^ expression.

Ago2 is the carrier of endogenous small interfering RNAs (endo-siRNAs) [15]. Although a cohort of endo-siRNA substrates have been identified, along with a number of targeted transcripts, the biological function of endogenous RNAi remains poorly defined. The major phenotypes are molecular in nature, and include upregulation of transcripts derived from transposable elements and certain direct targets of hairpin RNA-derived siRNAs [23], [24], [25], [26], [27], [28]. A detailed analysis of the true *ago2* null alleles *ago2^454^* and *ago2^321^* might therefore provide the most stringent test of the overall requirements of the endo-siRNA pathway for various aspects of development.

### GRR variation does not noticeably disrupt development

Our expression analysis indicates that *D. melanogaster* is able to produce Ago2 variants with and without the glutamine-rich NTD. This observation suggests that the NTD serves some important function as its expression is retained even though NTD-less forms can apparently be produced.

Our previous work had suggested a biological role for the NTD. We discovered variants in the GRR pattern and found that they were associated with severe disruption of embryonic development [16]. In particular, we had found that embryos from mothers homozygous for *dop* mutations show delays in cellularization, display abnormal transport of lipid droplets, and fail to hatch. Sequencing of genomic DNA of the *dop^1^* allele revealed that in this strain *ago2* has 4 copies of GRR1 and 10 copies of GRR2; in contrast, the precursor chromosome (*red e*) exhibited the expected 4xGRR1/11xGRR2 pattern of the published wild-type sequence. For an independently derived *dop* allele (*dop^46^*), *ago2* displayed 3xGRR1 and 11xGRR2. A functional connection between *dop* and *ago2* was suggested by the observation that a chromosomal deletion (*Df(3L)XG9*) uncovering *ago2* does not complement *dop* alleles and that the maternal lethality of *dop* mutations is partially rescued by expression of an *ago2* cDNA [16]. Finally, *dop* alleles genetically interact with other genes involved in RNA silencing. These results suggested that deviation from the wild-type number of GRRs severely impairs Ago2 functions and results in developmental defects [16].

**Figure 2:**
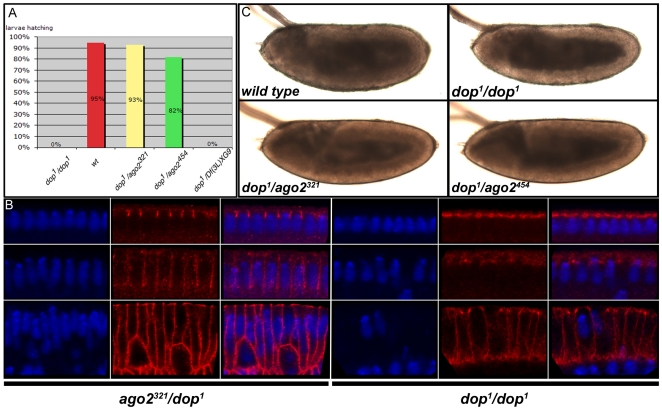
Null alleles of *ago2* complement *dop^1^* mutants. (**A**) Percentage of larvae hatching from indicated genotypes (n>300 per genotype). (**B**) Bright-field images of post-gastrula embryos (stage 10) show that the periphery of *dop^1^* mutant embryos is abnormally transparent compared to wild type (*wt*) [16]. These transparency changes are characteristic of altered lipid-droplet distribution [57]. Embryos obtained from mothers transheterozygous for *dop^1^* and either *ago2* ^321^ or *ago2* ^454^ exhibit a normal pattern lipid-droplet distribution. (**C**) Immunolabeling of the membrane protein Neurotactin (red) and DNA (DAPI, blue) in embryos during and after cellularization. Embryos from *dop^1^* homozygous females exhibit a severe delay in membrane ingression marked by the absence of Neurotactin staining at the interface between adjacent nuclei [16]. Embryos from *dop^1^/ago2^321^* transheterozygous mothers show normal membrane ingression during cellularization.

The identification of true null alleles of *ago2* (above) enabled us to perform a stringent genetic test of this idea. *dop* homozygous as well as *dop*/*Df(3L)XG9* mothers are completely sterile. However, mothers carrying one *dop* allele (either *dop^1^* or *dop^46^*) and one *ago2* null allele (*ago2^321^* or *ago2^454^*) were fertile. For example, the embryos obtained from *dop^1^*/*ago2^321^* heterozygotes hatched at rates similar to wild type (Fig. 2A).

The embryos from transheterozygous (*ago2^null^*/*dop*) mothers also did not exhibit the developmental defects characteristic of *dop* mutant embryos. Embryos obtained from *dop^1^* or *dop^46^* homozygous mothers show normal syncytial development, but plasma membrane growth during cellularization is strongly delayed (Fig. 2B; [16]). Embryos obtained from transheterozygous mothers do not display such delay in cellularization (Fig. 2B). Furthermore, lipid droplets are severely mislocalized in *dop* mutant embryos during germ-band extension (Fig. 2C). In contrast, lipid-droplet distribution in embryos from *ago2^null^*/*dop* mothers is similar to the wild type (Fig. 2C). These results demonstrate that *dop* is not allelic to *ago2.* In addition, there is no evidence that *ago2* with non-standard GRR pattern results in developmental defects.

### GRR variation does not affect RISC assembly

Depletion of Ago2 from cell lines or flies results in a complete block of the RNAi response [10]. Furthermore, biochemical data indicate that Ago2 is an essential and sufficient component of RISC, the central machinery that directs mRNA cleavage by siRNAs [10], [12], [29], [30]. Ago2^long^ is essential for this process, because *ago2^51B^* or *ago2^414^* homozygotes lack most, if not all, of the RNAi response [11], [16]. Since the *dop^1^* chromosome (characterized by the 4/10 GRR *ago2* pattern) exhibits a mild suppression of experimental RNAi in vivo [16], variation in Ago2^long^ might modulate the formation of RISC.

**Figure 3:**
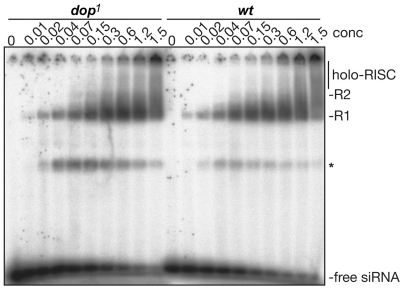
Comparison of RISC formation in wild type and *dop^1^* mutant embryos. In-vitro silencing complexes formation was analyzed by incubating dilutions of cell free extracts (conc: µg/µl total protein) of *wild type* or *dop^1^ Drosophila* embryo extracts with pre-labelled siRNA. The formed silencing complexes were separated on a native acrylamide gel. The free-siRNAs and the R1, R2 and holo-RISC complexes are shown (The star marks an unknown band).

To test this possibility directly, we assayed RISC-formation in vitro in embryo extracts obtained from wild-type and *dop^1^* embryos, using gel shift experiments with siRNA [31]. RISC formation as observed by assembly of complexes over time was unimpaired in *dop^1^* mutant embryos when compared to wild type (Fig. 3). We therefore conclude that the variation of GRR pattern in *dop* mutants does not affect formation of RISC. This finding is consistent with reports that recombinant Ago2 lacking the first 277 aa of the NTD (this truncation removes all GRR1 and seven GRR2 repeats) supports RISC assembly [19].

### GRR copy number varies extensively between different fly strains

The complementation tests above demonstrate that *dop* and *ago2* represent distinct genes. Yet, in two independently derived lines, *dop* mutations are associated with changes in the GRR pattern of *ago2* [16]. This fortuitous association might be explained if changes in the GRR pattern of *ago2* occur at high frequency.

**Figure 4:**
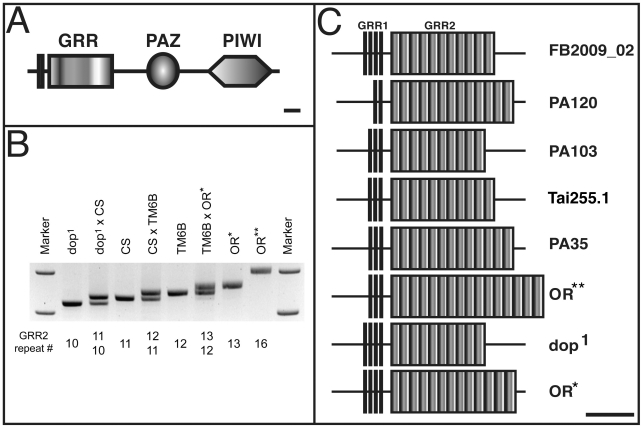
Different Ago2 GRR haplotypes in *Drosophila melanogaster*. (**A**) The domain structure of Ago2_PB protein isoform, according to FlyBase version FB2009_2. The amino-terminal half contains glutamine-rich repeats (GRR), shown in more detail in (**C**). The carboxy-terminal half contains the PAZ and the PIWI domains (bar indicates 100 amino acids). (**B**) The copy number of GRR2 repeats varies between various laboratory strains. Using flanking primers, the genomic region encompassing the GRR2 repeats was amplified by PCR in various laboratory strains, or in the progeny of crosses between those strains. *CS* =  *Canton S*; *TM6B*  =  balancer chromosome (tested over a deficiency encompassing the *ago2* locus); OR* and OR** are *Oregon R* strains obtained from two independent sources. Below the gel image is the number of GGR2 repeats as determined by sequencing. (**C**) Different *ago2* haplotypes determined by genomic DNA sequencing (see Table 1 for a complete list of all haplotypes determined in this study).

We therefore examined GRR1 and GGR2 copy number in twenty-two laboratory strains of diverse origins. In these strains, GRR1 copy number varied between 2 and 4, and GRR2 copy number between 10 and 16 (Table 1, Fig. 4, Fig. S1, and data not shown). For example, copies of the wild-type strain *Oregon R* obtained from two different laboratories had 4x GRR1 and 13x GRR2 or 3x GRR1 and 16x GRR2, respectively. Various combinations of GRR1 and GRR2 copy numbers can occur. In the following, we refer to these combinations as GRR haplotypes.

**Table 1:** GRR pattern in Ago2 in different *D. melanogaster* strains.

Line	GRR1	GRR2	Length of NTD	Glu in NTD	% Glu in NTD
*3CPA120*	2	13	431	168	39%
*3CPA6*	2	[10]			
*3CPA103*	3	10	368	146	40%
*3CPA54*	3	[11]			
*3CPA35*	3	[13]			
*3CPA129*	3	n.d.			
*3CPA122*	4	11	397	159	40%
*3CPA31*	4	[11]			
*3CPA113*	4	[11]			
*3CPA2*	4	[12]			
*Canton-S*	n.d.	[11]			
*w-14 melbourne*	4	11	397	159	40%
*Tai255.1*	3	11	391	155	40%
Or R*	4	13	443	177	40%
Or R**	3	16	506	199	39%
*dop* [*46*]	3	11	391	155	40%
*dop* [*1*]	4	10	374	150	40%
*red e*	4	11	397	159	40%
*In(1)AB*	4	11	397	159	40%
*TM6B*	4	12	420	168	40%
*TM8*	n.d.	[11]			
*TM1*	n.d.	[12]			
*LVM*	n.d.	[12]			
*Dp(3;3)Cam36*	n.d.	[13]			

Genomic DNA encompassing the GRR1 or GRR2 repeats was amplified using PCR. The number of repeats was estimated by gel electrophoresis as in Fig. 3A (numbers in brackets) or determined by sequencing the PCR fragments (Fig. S1). Top: The *3CPA* strains were derived from a single wild North American population in 2001 [32]. Middle: Wild-type laboratory strains; note that two strains characterized as *Or R* (obtained from different laboratories) had distinct GRR repeat patterns. Bottom: A selection of other laboratory strains; homozygous lethal chromosomes (*LVM*, *TM6B*, *TM1*, *Dp(3;3)Cam36*) were analyzed as transheterozygotes with the deficiency *Df(3L)XG9* that encompasses the *ago2* locus.

To assess if variation in GRR copy number is a consequence of extended laboratory culture, we examined ten strains recently isolated from the wild. These strains are isogenic for third chromosomes that had been extracted from a single wild North American population [32]; we analyzed their GRR haplotypes within five years after they had been established. These strains exhibited GRR haplotype diversity equal to or greater than that found in the long-established laboratory strains (Table 1).

Our results indicate that in *D. melanogaster* the NTD of Ago2 can undergo drastic changes in length and sequence composition. As we have not attempted a comprehensive survey, we suspect that GRR variation is not restricted to the haplotypes we identified. Considering just the instances listed in Table 1, NTD length can vary by over 35% (from 368 to 506 amino acids). These changes in the NTD likely arise by repeat expansion and contraction mediated by unequal crossing over [33]. Interestingly, the glutamine fraction of the NTD remains fairly constant (∼40%).

### Failure to detect phenotypic consequences of NTD variability

**Figure 5:**
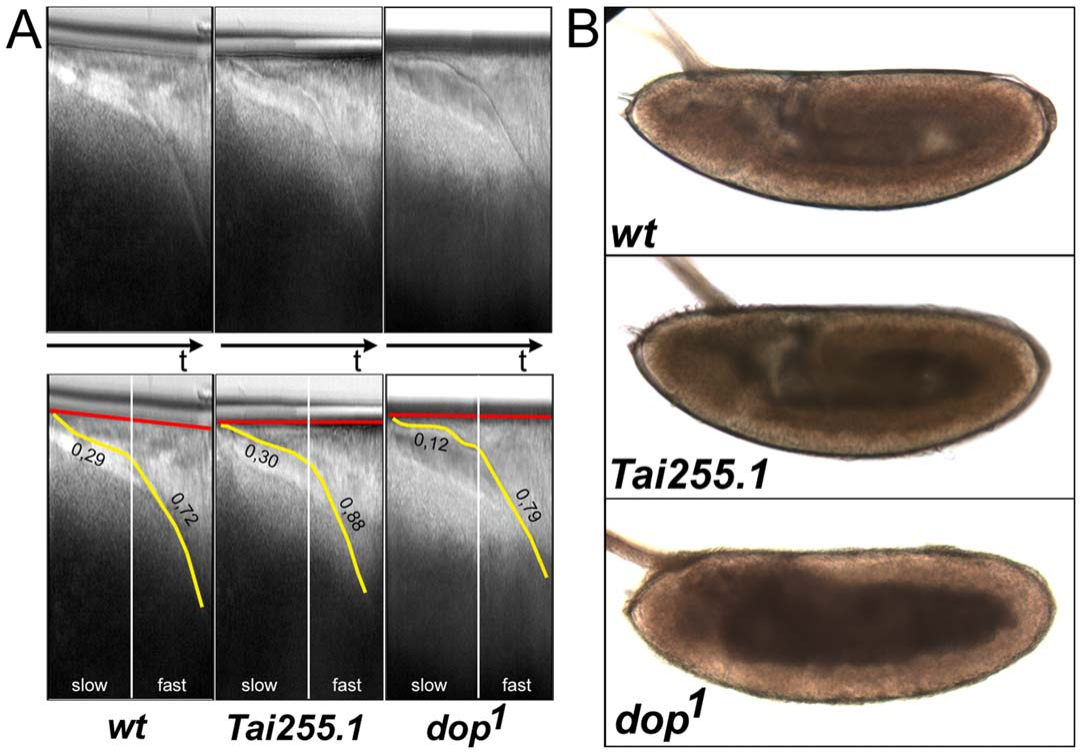
Altered GRR pattern is not associated with developmental defects characteristic of *dop* alleles. (**A**) Kymographs from time-lapse microscopy of *w^1118^*, *Tai255.1* and *dop^1^* embryos. Upper panels show primary data and lower panels indicate the embryo surface membrane (red) and the ingressing plasma membrane (yellow) over time (t). In the wild type (wt), membrane ingression is initially slow at 0.29 µm/min) and then increased in the second phase to 0.72 µm/min. Similar values were observed for *Tai255.1*. *dop^1^* mutant embryos exhibit a strong reduction in membrane ingression during slow phase (0.12 µm/min); fast phase is normal. (**B**) Bright-field images of post-gastrula stage embryos indicating distribution of lipid droplets in wild-type (wt), *Tai255.1* and *dop^1^* mutant embryos. In wild-type and *Tai255.1* embryos, the periphery of the embryo is opaque due to the presence of lipid droplets within the ectoderm and mesoderm cell layer. *dop^1^* mutant embryos exhibit a characteristic lipid-droplet transport defect indicated by a concentration of lipid droplets in the center of the embryo and a transparent embryonic periphery.

The NTD variability we uncovered provides an independent test whether GRR copy number and the *dop* phenotype are linked. Many of the strains in Table 1 are homozygous viable and at least grossly normal, suggesting that the exact composition of the amino-terminal domain of Ago2 is nonessential for viability and that many GRR haplotypes are compatible with normal development. In particular, the *Tai255.1* strain, a wild-type *D. melanogaster* strain isolated 1983 at Ivory Coast [34] had the same 3/11 GRR haplotype found in the *dop^46^* allele. However, unlike *dop^1^* or *dop^46^* embryos, *Tai255.1* embryos develop apparently normally (Fig. 5). They cellularized normally and displayed normal lipid-droplet transport (Fig. 5A,B). We therefore conclude that GRR variation in *ago2* does not disrupt embryogenesis and in particular is not responsible for the developmental defects observed in *dop* mutants.

**Figure 6:**
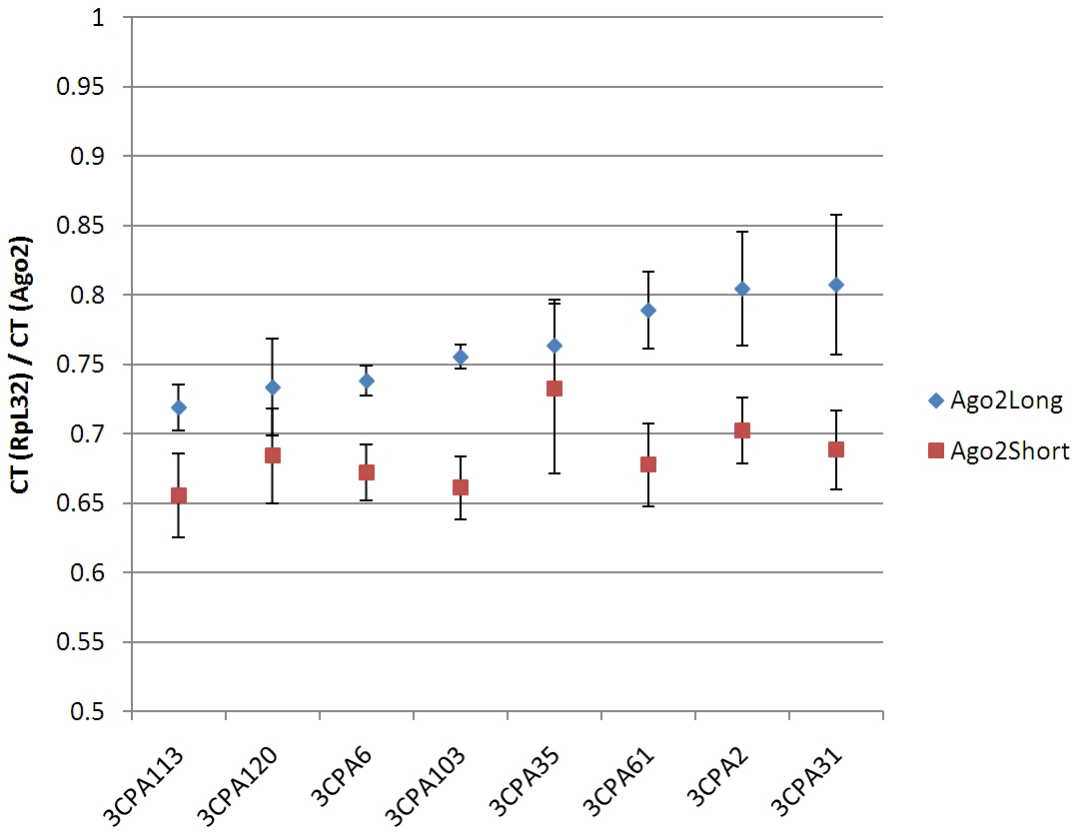
Expression of *ago2* ^short^ and *ago2* ^long^ in wild-type *D. melanogaster* strains. Levels of *Ago2^long^* (blue) and *Ago2^short^* (red) in the 3CPA strains. Each symbol represents mean expression level relative to *RpL32*, indicated as ratio of qtPCR critical thresholds (“CT”). Bars represent one SEM. X axis denotes strain.

We next examined if GRR variation influences expression of long and short Ago2 isoforms. Eight wild type third-chromosome extracted 3CPA strains all express both long and short *ago2* transcripts (Fig. 6). We conducted quantitative real-time PCR using *ago2* long- and short-specific primers and cDNA prepared from 3-day old females [35]. *ago2* ^long^ expression varied significantly among the strains (ANOVA; *p*<0.05), and was consistently higher than *ago2* ^short^ expression (Fig. 6; average *ago2* ^long^ expression is 11% higher than *ago2* ^short^). In contrast, *ago2* ^short^ expression did not vary significantly among the strains, and we detected no correlation between *ago2* ^long^ and *ago2* ^short^ expression levels. Last, there was no obvious relationship between GRR haplotypes and *ago2* ^long^ or *ago2* ^short^ expression in these strains. These results suggest independent regulation of transcripts for *ago2* ^long^ and *ago2* ^short^.

In summary, we find no evidence that GRR variation is associated with dramatic changes in embryonic development or survival, in RISC formation or with changes in isoform expression. It is conceivable that GRR variation does not have any functional consequences and simply arises as “noise” due to the propensity of repeat regions to undergo unequal crossing over. Alternatively, GRR variation may be involved in more subtle functions of Ago2 than the ones tested here. While RISC formation per se can occur in the absence of most of the GRRs [19], regulation of Ago2 activity or efficient recognition of certain targets might be modulated by the NTD. For example, the long Ago2 isoform, which includes the NTD, is implicated in anti-viral responses as the *ago2^414^* allele, which still expresses the short Ago2 isoform (above), exhibits strongly impaired host defense against viruses [7]. Future population-genetic and functional tests of Ago2 should therefore examine a range of complete *ago2* haplotypes or, at a minimum, carefully define which “wild-type” version of Ago2 is being analyzed.

### Extensive remodeling of the NTD between closely related species

Previous studies have uncovered unusually high variability in non-NTD regions of Ago2 [36], [37]. Comparison of the C-terminal 839 amino acids of Ago2 (i.e. largely excluding the NTD) in pairs of recently diverged *Drosophila* species revealed rapid evolutionary changes. These observations placed *ago2* among the fastest evolving genes in Drosophilids, along with other components of the RNAi pathway [36]. It was proposed that this reflects adaptive evolution in response to changes in viral pathogens, as part of a virus-host arms race [37]. The observed changes in these RNAi pathway genes indeed bear the signature of positive selection [36].

The previously described evolutionary changes in Ago2 are concentrated in regions of unknown function [36]. In contrast, regions of Ago2 associated with core functions are conserved across all *Drosophila* species. The function of the NTD is also unknown. We therefore examined the NTDs in closely related species to determine whether their sequence evolution matches the former or latter pattern.

For this analysis, we focused on the three species most closely related to *Drosophila melanogaster*, namely *Drosophila simulans*, *Drosophila mauritiana*, and *Drosophila sechellia*. These species diverged <1 Mya (*sechellia* versus *simulans*) or ∼2.3 Mya (*D. melanogaster* versus *D. sechellia*/*simulans/mauritiana* lineages) ago from each other, and the sequences of most genes are highly conserved in all four species [38], [39].

We first examined the DNA sequences that correspond to exon 3 of *ago2* in *D. melanogaster*. Here, this exon encodes 330 amino acids of the NTD (including all GRR2 repeats) plus the following 92 amino acids, whose sequence is conserved in Argonautes from fungi, plants and animals. The latter, non-NTD stretch displayed very high amino acid sequence conservation between the four *Drosophila* species (Fig. S2, S3, and data not shown). And in each of the four species, the sequence immediately 5′ to this conserved region has the potential to encode glutamine-rich, repetitive stretches, but the exact sequence is highly divergent ([16], and Fig. S2). For example, the 23 aa GRR2 repeats characteristic of *D. melanogaster* are not apparent in any of the other three species. This observation suggests that NTDs can change drastically between even closely related species.

As the Ago2-encoding genomic sequences of *D. simulans* and *D. mauritiana* were nearly identical but differed from both *D. sechellia* and *D. melanogaster*, we performed a detailed comparison of the entire NTD between *D. melanogaster*, *D. simulans* and *D. sechellia*. The analysis above does not reveal the full extent of the NTDs or if these putative NTDs are indeed expressed. We therefore predicted possible upstream exons and designed primers in these exons and in exon 5 for RT-PCR analysis. For both *D. simulans* and *D. sechellia*, this strategy allowed us to amplify cDNA fragments spanning these exons; sequencing revealed that these fragments indeed encompass the putative exon 3, including the highly variable glutamine-rich stretches. Thus, like *D. melanogaster*, these species express unusual Ago2 NTDs.

The NTDs of the three species differ extensively from each other in length (*D. sechellia*: 211 aa; *D. simulans*: 277 aa; *D. melanogaster* 368 to 506 aa (Table 1)); their glutamine content, however, was fairly similar (36–40%) (Table 2; Fig. S3). These length differences do not arise from variation in copy numbers of similar repeats, as between distinct *D. melanogaster* strains (Table 1). Rather, NTD organization is quite different between species. The primary sequences of the *simulans* and *sechellia* NTDs are clearly related; both species contain a highly similar sequence of ∼118 aa. But *D. simulans* Ago2 has imperfect, tandem copies of this sequence while *D. sechellia* Ago2 has a single copy (Fig. S2B). Instead, in *D. sechellia* Ago2, a 15 aa stretch internal to the 118 aa region is repeated three times. Neither the 118 aa nor the 15 aa repeat displays striking similarity to the 6 aa (GRR1) or 23 aa (GRR2) repeats in *D. melanogaster*. In contrast, the remainder of the Ago2 sequence in all three species is highly similar and can easily be aligned (Fig. S3). Thus, the primary sequence of the NTD is extremely variable, much more so than the rest of the protein (which already is among the most rapidly evolving proteins in *Drosophila* [36]).

**Table 2:** NTD of Ago2 orthologs in *Drosophila* species.

species	name	Length of NTD	Glu in NTD	% Glu
*melanogaster*	Dmel\Ago2-PC	397	159	40%
*melanogaster*	Dmel\Ago2^short^	0	0	N.A.
*simulans*	Dsim\Ago2	277	101	36%
*sechellia*	Dsec\Ago2	211	83	39%
*yakuba*	Dyak\Ago2	357	153	43%
*erecta*	Dere\Ago2	298	124	42%
*ananassae*	Dana\Ago2	214	78	36%
*pseudoobscura*	Dpse\Ago2a	226	43	19%
*pseudoobscura*	Dpse\Ago2b	182	36	20%
*pseudoobscura*	Dpse\Ago2c	157	55	35%
*pseudoobscura*	Dpse\Ago2d	149	34	23%
*pseudoobscura*	Dpse\Ago2e	46	3	7%
*persimilis*	Dper\Ago2a	5	0	0%
*persimilis*	Dper\Ago2b	200	48	24%
*persimilis*	Dper\Ago2c	142	57	40%
*persimilis*	Dper\Ago2d	142	34	24%
*persimilis*	Dper\Ago2e	46	3	7%
*willistoni*	Dwil\Ago2a	202	56	28%
*willistoni*	Dwil\Ago2b	190	51	18%
*mojavensis*	Dmoj\Ago2	247	57	23%
*virilis*	Dvir\Ago2	335	96	29%
*grimshawi*	Dgri\Ago2	316	99	31%

Predicted Ago2 proteins in the 12 sequenced *Drosophila* species were examined for length and glutamine content of the amino-terminal domain. The NTD was defined by comparison to *Homo sapiens* Ago2, as described in Experimental Procedures. For details of how these proteins were predicted and for FlyBase or GenBank names, see Fig. S4. For *D. melanogaster*, the canonical (3xGRR1, 11xGRR2) long isoform and the short isoform are listed.

In *D. melanogaster*, the NTD displays drastic intra-specific variation (Table 1). To test if similar variability might occur in *D. simulans*, we amplified by PCR the genomic region corresponding to exon 3 from nine different strains and sequenced the PCR fragments. In total, we recovered five haplotypes that vary from each other by small deletions and insertions (Fig. S2A). We conclude that the NTD evolves rapidly both within and between species.

### The NTD of Ago2 is highly variable throughout insects

**Figure 7:**
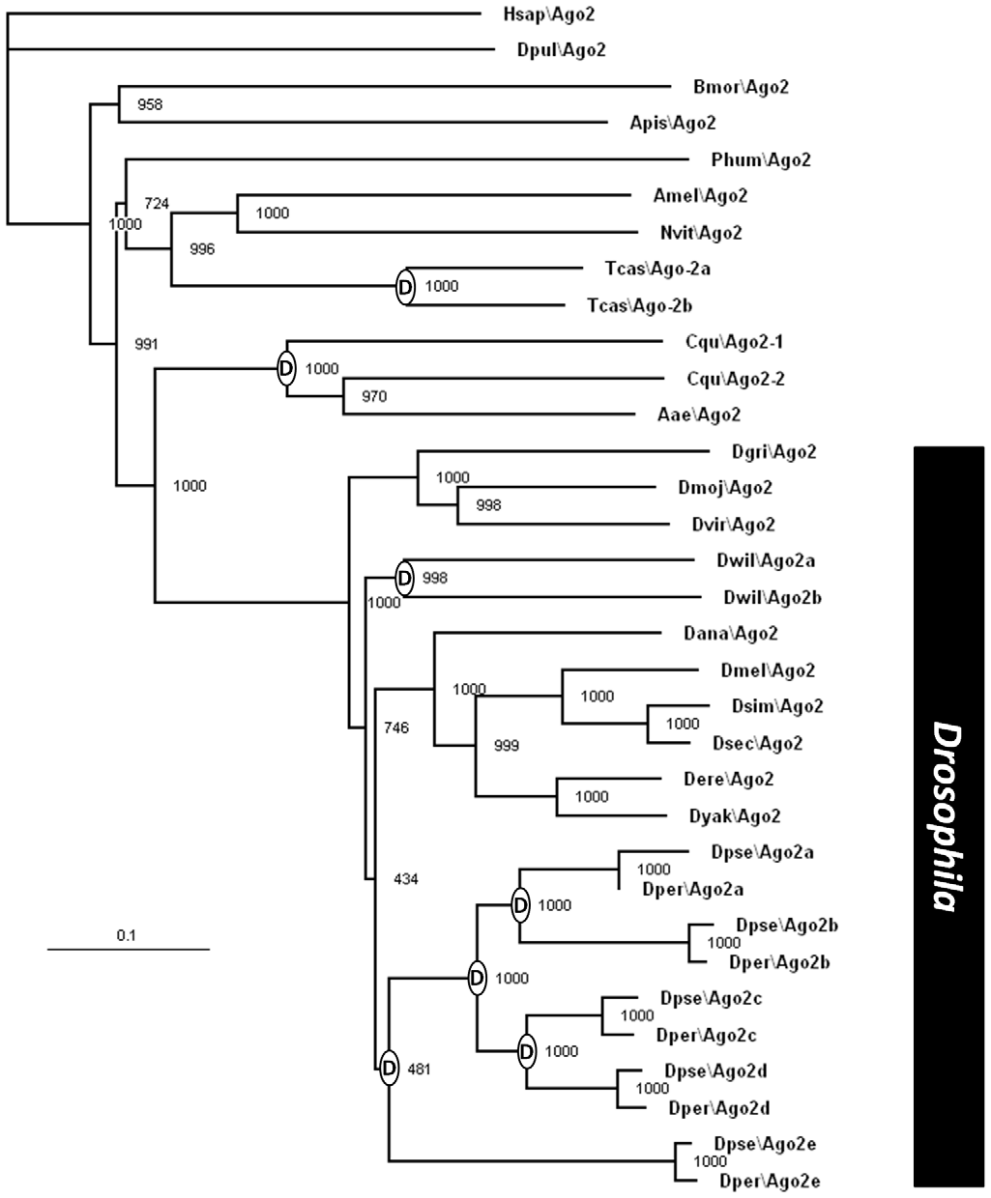
Rooted neighbor-joining phylogram of Ago2 proteins. Tree is rooted with *Homo sapiens* Ago2. Branch lengths (genetic distance) indicated by ruler. Numbers at nodes indicate supportive number of 1000 bootstrap trials. Nodes labeled with D indicate gene duplications. The genus *Drosophila* is indicated by a black bar. Species designations: Hsap, *Homo sapiens;* Dpul, *Daphnia pulex*; Bmor, *Bombyx mori*; Apis, *Acyrthosiphon pisum*; Phum, *Pediculus humanus*; Amel, *Apis mellifera*; Nvit, *Nasonia vitripennis*; Tcas, *Tribolium castaneum*; Cqu, *Culex quinquefasciatus*; Aae, *Aedes aegypti*; Dgri, *Drosophila grimshawi*; Dmoj, *D. mojavensis*; Dvir, *D. virilis*; Dwil, *D. willistoni*; Dana, *D. ananassae*; Dmel, *D. melanogaster*; Dsim, *D. simulans*; Dsec, *D. sechellia*; Dere, *D. erecta*; Dyak, *D. yakuba*; Dpse, *D. pseudoobscura*; Dper, *D. persimilis*.

In the genus *Drosophila*, nearly full genome sequences are available for nine additional species [40]. This resource provides an opportunity to examine the pattern of Ago2 evolution across longer evolutionary times, on the order of 50 Mya. We first identified all Argonaute family members in the predicted proteomes of all twelve species, by reciprocal blastp best-hit analysis and comparison of FlyBase annotations in all 12 species. We conducted multiple alignment of the protein sequences, neighbor-joining tree building and 1000 bootstrap trials via CLUSTALX (data not shown). Examination of the phylogram revealed a subset of these proteins as most closely related to Ago2 of *D. melanogaster*, clearly separate from Ago1, Piwi, Aubergine, and Ago3 orthologs. Interestingly, *D. willistoni* had two Ago2 paralogs and *D. pseudoobscura* and *D. persimilis* each had five Ago2 paralogs (see also Fig. 7). Multiple Ago2s in a single genome have also been reported for the mosquito *Culex quinquefasciatus* [41] and the flour beetle *Tribolium castaneum* [42] (Table 3). Presumably, multiple *ago2* genes in a single organism allow those variants to be adapted for specific tasks. We speculate that in *D. melanogaster* the multiple isoforms generated from a single *ago2* gene allow similar specialization.

**Table 3:** NTD of Ago2 orthologs in other species.

species	name	Length of NTD	Glu in NTD	% Glu
*Culex quinquefasciatus*	Cqu\Ago2-1	87	20	23%
*Culex quinquefasciatus*	Cqu\Ago2-2	208	41	20%
*Aedes aegypti*	Aae\Ago2	173	77	45%
*Apis mellifera*	Amel\Ago2	366	148	40%
*Nasonia vitripennis*	Nvit\Ago2	222	50	23%
*Bombyx mori*	Bmor\Ago2	232	11	5%
*Tribolium castaneum*	Tcas\Ago2a	49	5	10%
*Tribolium castaneum*	Tcas\Ago2b	69	18	26%
*Acyrthosiphon pisum*	Apis\Ago2	146	26	18%
*Pediculus humanus*	Phum\Ago2	429	92	21%
*Daphnia pulex*	Dpul\Ago2	272	67	25%
*Homo sapiens*	Hsap\Ago2	24	1	4%

Predicted Ago2 proteins in various insects and the crustacean *Daphnia pulex* were examined for length and glutamine content of the amino-terminal domain, using the same approach as for Table 2. Human Ago2 is included for comparison.

The protein annotation for the 11 non-*melanogaster* genomes employs a consensus between eight distinct gene prediction algorithms [40]. For most of the Ago2 candidates, either this consensus or at least one of the individual algorithms indicated the presence of a glutamine-rich NTD, suggesting that such amino-terminal domains are common throughout the genus. In total, 18 out of the 21 Ago2 sequences in Table 2 have an NTD of more than 240 amino acids and an NTD glutamine content above 12%. Although the presence of these NTDs will have to be independently confirmed in the future, for several of these candidates, cDNA or microarray evidence support that the NTD region is indeed expressed (Table S1).

Although many of these predicted Ago2 proteins have glutamine-rich NTDs, the primary sequence, length and glutamine content vary greatly between family members (Table 2, Fig. S4). This is even true for Ago2 paralogs in the same species, such as for the Ago2s of *D. willistoni* that have NTDs of 244 and 356 amino acids, respectively. And some of the *D. persimilis* and *D. pseudoobscura* copies even seem to lack a glutamine-rich NTD entirely. In addition, there are radical changes in repeat organization between species [16]. For example, in various Ago2s, we identified repeats of 11, 12, 13, 16, 22 and 31 amino acids in length. Even in the sister species *D. persimilis* and *D. pseudoobscura* which diverged ∼0.5 Mya ago [43], the closely related Ago2 pair Dpse\Ago2c and Dper\Ago2c are identical across much of the NTD and the rest of the coding sequence, but are distinguished from each other by unique repeats (Fig. S5).

To determine if Ago2 NTDs are found outside the genus *Drosophila*, we searched various databases for Ago2 candidates and identified them in a wide variety of insect species, including flies, beetles, bees, wasps and lice, and even the crustacean *Daphnia pulex* (Fig. S4). A comparison with Argonaute family members from fungi, plants and animals (i.e., the dataset analyzed in [17]) indicated that insect plus *Daphnia* Ago2s are a monophyletic assemblage, well supported by bootstrap trials (data not shown). As previously noticed for *D. melanogaster* Ago2 [17], this entire clade is highly divergent and distinct from all other Argonautes. And like the *Drosophila* Ago2s, most members of this clade are predicted to carry glutamine-rich amino-terminal domains ([16], Fig. 7, Fig. S4, Table 3). In several instances, cDNA data support that these NTDs are expressed (Table S1). These domains are often, but not always, organized as multiple copies of imperfect repeats. As in the genus *Drosophila*, the lengths and sequences of these repeats vary substantially ([16], Fig. S4), and the NTDs overall do not display extensive similarity in primary sequence. Thus, highly variable Ago2 NTDs are apparently ancient and predate the origin of insects.

At the moment, it is impossible to determine whether drift or selection is the primary determinant of this variability. It is conceivable that the dramatic changes in NTD primary sequence are simply a consequence of the high instability of repetitive DNA sequences. If the NTD has no function, it may be free to vary with little constraints. In this scenario, selective sweeps due to positive selection on the rest of the Ago2 sequence, *e.g.* as frequency-dependent adaptation of the anti-viral response [36], may cause certain NTD haplotypes that happened to be present in those *ago2* variants to rise in frequency via hitchhiking. Indeed, the number and heterozygosity of NTD alleles in the natural PA population is striking, especially when compared with other protein-coding repeats which may experience similar selective pressures. Considering the unique GRR1/GRR2 haplotypes listed in Table 1 (excluding missing data), there are 7 NTD alleles segregating among the PA strains. Heterozygosity, measured as one minus the sum of the allele frequencies, is 0.815. A recent survey of 58 protein-coding repeats in the same strains found lower diversity overall [44] the mean and maximum number of alleles were 2.83±0.2 and 8, respectively. Mean and maximum heterozygosity in the same survey were 0.457±0.03 and 0.83. This survey considered primarily homotypic amino-acid repeat loci, not mixed or “minisatellite” repeats as are found in the Ago2 NTD. Analyses of mixed protein-coding repeats in natural *D. melanogaster* populations are less common. The best-studied example involves the period (*per*) gene, which harbors a Thr-Gly repeat of varying length that is subject to strong natural selection imposed by photoperiod and climate [45]. A large survey of Australian populations found 8 *Per* Thr-Gly alleles segregating, with overall heterozygosity of 0.640 [46]. Thus, Ago2 NTD intraspecies variability is among the highest measured for protein-coding repeats in *D. melanogaster,* with very high heterozygosity consistent with selection-mediated maintenance of allele frequencies. Unlike *per*, however, no clear phenotypic basis for selection on NTD alleles is evident. To determine whether and how selection operates on the NTD *vs.* linked Ago2 regions, future studies should survey natural variability and linkage disequilibrium throughout the entire *ago2* locus. Such studies will greatly benefit from recent efforts to sequence multiple *D. melanogaster* genomes.

Glutamine-rich NTDs of low sequence complexity are present across many insect species, even in species that are apparently able to generate Ago2 variants lacking the NTD (such as Ago2^short^ in *D. melanogaster* and Ago2e in *D. persimilis* and *pseudoobscura*). We therefore favor the hypothesis that the NTD provides an important regulatory activity. Given the role of Ago2 in the defense against viruses [7], [9], it is an intriguing possibility that NTD variability modulates anti-viral responses. It will be revealing to test if the survival after viral challenge is different in *D. melanogaster* strains expressing endogenous Ago2 or Ago2 variants in which the NTD is deleted or replaced by the NTD of other insect species.

How the NTD might modulate the biological function of Ago2 is an exciting question for the future. A possible paradigm comes from the analysis of *Trypanosoma brucei* Ago1: Here, the amino-terminal 68 amino acids contain ten arginine-glycine rich motifs [47]. This so-called RGG domain is involved in the association of TbAgo1 with poly-ribosomes, an association required for an efficient RNAi response in this organism [48]. Intriguingly, the TbAgo1 RGG domain and the NTDs of arthropod Ago2s are all characterized by low sequence complexity and are likely to be intrinsically unstructured (based on RONN algorithm predictions ([49], data not shown). As intrinsically unstructured domains have been proposed as protein interaction domains [50] and changes in the number of glutamine-rich repeats can modulate interaction strength [51], [52], RGG or NTD variation might modulate the affinity of the Argonaute protein to particular targets. Indeed, mutant versions of TbAgo1 show progressively stronger RNAi responses, the more copies of the RGG motif are present [48].

### Conclusion

The evolution of the Argonaute protein family resulted in members with specific tasks in small RNA functions within a single organism. Here we uncover additional complexity: Many insects express multiple Ago2 variants, either from independent genes or by generating multiple isoforms from a single gene. In addition, there is variation between different individuals of the same species, due to extensive variation in NTD organization. The functional significance of this variation remains unknown; we find that it does not grossly alter embryonic development or RISC assembly. But given the known role of Ago2 in protection against viruses, we speculate that this variability might allow fine-tuning of anti-viral responses. Such a modulatory role of the NTD would also be consistent with the rapid evolution of this domain, as observed in comparisons across insects. A potential association between NTD haplotype and Ago2 activity might provide novel insights into the plasticity of RNAi pathways in general and the innate viral response mechanisms in particular.

## Materials and Methods

### *Drosophila* strains and culture

*Drosophila* strains and embryos were cultured under standard laboratory condidtions. The following strains were used in this study: Oregon R, Canton-S (gift from L. Griffith), *ago2* ^51B^ (gift of F.-B. Gao), *ago2* ^414^, *ago2* ^454^, *ago2* ^321^. Screening of deletion mutants was carried out as previously described [11]. The EP transposon in the *EP(3)3417* line was mobilized by crossing *EP(3)3417* virgins with males possessing *Δ2-3* transposase. Deletions were determined as described previously [11]. The *D. melanogaster 3CPA* strains were donated by B.P. Lazzaro and had been established in 2001 via extracting 3rd chromosomes segregating in a single Pennsylvania (USA) population over balancers and crossing into a common background [32]. *D. melanogaster strains Tai255.1*, *w-14* melbourne and Oregon R** as well as *D. simulans* strains *Oxnard*, *Tsimbazaza*, *vermilion*, *maz1*, *maz6* and *C167.4* were a gift from H. Hollocher. These strains are described in Sainz et al. [53]. Additional strains were obtained from the Tucson *Drosophila* Stock Center: three *D. simulans* strains (*sim1* =  #14021-0251.48, *sim2* =  #14021-0251.047, *sim3* =  #14021-0251.004), one *D. mauritiana* strain (#14021-0241.46) and two *D. sechellia* strains (#14021-0248.08, #14021-0248.25). All other strains in Table 1 were obtained from the Bloomington *Drosophila* Stock Center.

### Quantitative real-time PCR

Groups of 15 female flies aged 3-5 days were anesthetized under light CO_2_ and flash-frozen in liquid N_2_. Total RNA was prepared from each group, and cDNA was synthesized using oligo-dT primers according to standard protocols (see Bettencourt *et al.* 2008). Three replicate preparations were obtained for each of the *3CPA* strains in Figure 6. QrtPCR was conducted using primers specific for *ago2* ^long^ and *ago2* ^short^; sequences are available upon request. The ribosomal protein gene *RpL32* was also examined as a control according to Bettencourt *et al*. (2008). A minimum of two replicate PCR reactions per strain/gene were conducted. Reactions were conducted and analyzed on a BIO-RAD myIQ thermocycler using a standard 40-cycle protocol, and amplification products were verified via electrophoresis and melt-curve.

### In vivo observations and immunohistology

For in vivo observation, embryos were collected and staged on yeasted apple juice plates and mounted in halocarbon oil 27 (Sigma-Aldrich) on microscope slides. Video-microscopy was performed on a Zeiss (Germany) microscope equipped with Nomarski optics, and time-lapse movies were taken using the OpenLab software (Improvision, UK). For immunohistology, embryos were either heat-fixed or fixed using modified Stefanini's fixative and stained with antibodies essentially as described elsewhere [54]. The following antibodies were used: mouse anti Neurotactin (DSHB; Iowa, USA); mouse anti Arm [55]. All secondary fluorochrome conjugated antibodies were from Dianova (Germany) or Molecular Probes (Eugene, OR). Imaging was performed on a Leica SP2 Confocal microscope, and image processing was performed with Adobe Photoshop.

### Molecular Biology

Molecular cloning was performed following standard protocols. RT-PCR was performed using ‘one Step RT PCR kit’ (Qiagen, Germany) with embryonic poly A^+^ RNA as templates. Primer sequences are available upon request. Products from RT-PCR were confirmed by sequencing (Seqlab, Göttingen, Germany). GenBank accession numbers of full-length cDNA for *D. melanogaster ago2* are BT003546 (EST RE04347, RB isoform) and BT099682 (EST RE36670, RC isoform). In addition, the sequence of the EST GM07030 (GenBank accession number AY094751) is consistent with expression of the *ago2* ^short^ isoform. PCR to analyze NTDs in *D. melanogaster*, *simulans*, *mauritiana* and *sechellia* was performed with primers located in introns surrounding exons 2 and 3 of *ago2*, respectively. Protein extraction and immunoblotting was performed as described before [16].

### Northern blotting

RNA samples were prepared from 2-4-day old male flies using Trizol. 10 µg total RNA was loaded in each lane on a denaturing agarose gel and transferred to Genescreen plus membrane. The template for the probe was prepared by PCR using an *ago2* cDNA fragment cloned in pGEMT-easy vector. The primers used for PCR amplification are available upon request. Probes were labeled using Random Primed DNA Labeling Kit (Roche) and P^32^-dCTP, and hybridized at 65°C in 500 mM Church phosphate buffer containing 7% SDS and 1 mM EDTA.

### RISC formation assay

*Drosophila* embryo extract preparation and synthetic siRNA labelling and annealing were described previously [56]. Reactions used different dilutions of cell free extraxts of wild-type or *dop^1^* mutant embryos, 5 nM of P^32^-end-labelled siRNA and 1x lysis buffer containing 10% v/v of glycerol in a total volume of 10 µl. Reactions were incubated for 30 min to allow silencing complex formation. Native gel electrophoresis for separation of silencing complexes was essentially as described previously [31]. The in vitro reaction mixtures were diluted with 10 µl of loading buffer (1x lysis buffer, 6% Ficoll 400), and part of the sample was analyzed on a 3.9% (39∶1 acrylamide-bisacrylamide) native acrylamide gel in 1xTBE buffer. The gels were dried and exposed to a storage phosphor screen.

### Ago2 sequence comparisons

For various species, Ago2 family members were identified by searching FlyBase annotations or GenBank entries. For Ago2 proteins from *Drosophila* species, most candidates showed a glutamine-rich NTD. For those candidates that did not, we examined the eight distinct gene prediction algorithms from which the FlyBase consensus prediction is derived [40]. In several additional cases, at least one of these algorithms predicted a glutamine-rich NTD (details provided in Fig. S4). DUF1785, PAZ and PIWI domains were estimated using the Conserved Domain Search at NCBI. To estimate the extent of the NTD, the candidates were aligned with human Ago2 using the “Align two sequences by using BLAST” function at NCBI; the NTD was defined as those sequences amino-terminal to where significant alignment was detected. Similar results are obtained when candidates are aligned to any of the other human Argonaute family members (not shown). For human Ago2, the NTD was defined by aligning to *D. melanogaster* Ago2. For the phylogram in Fig. 7, we conducted multiple alignment of the protein sequences, neighbor-joining tree building and 1000 bootstrap trials via CLUSTALX. In similar phylograms based on all *Drosophila* Argonaute family members (not shown), the branch lengths for Ago2s are much longer than for Ago1s, consistent with the observation that Ago2 evolves much more rapidly than Ago1 [36]. These increased branch lengths hold true even if the NTD sequences of Ago2 are omitted from the analysis.

## Supporting Information

Figure S1 **Amino acid sequence of GRR2 repeats in various** ***D. melanogaster*** **strains.** GRR2 repeats display slight sequence variations, indicated by different colors. Fly strains differ both in number and type of repeat. The *3CPA122* pattern is identical to the one in the current FlyBase annotation for Ago2, and was also found in strains *In(1)AB*, *w-14 melbourne*, *red e*, and *dop46*. Note that the same overall repeat copy number can be achieved via distinct primary sequences (compare left and right columns below). This variability in sequence likely results in even greater number of distinct haplotypes, and the ten haplotypes in Table 1, which are based solely on repeat copy number, probably underestimate the true variability among the 32 strains surveyed.
(PDF)

Figure S2 **Variability in the Ago2 NTD for** ***Drosophila simulans*** **,** ***mauritiana*** **, and** ***sechellia*** **strains.** The genomic DNA corresponding to exon 3 of *ago2* was sequenced in a number of strains of the *D. simulans* species complex. The amino acid sequences for the corresponding NTD stretches were compared. A) Nine *D. simulans* and one *D. mauritiana* strain displayed highly similar sequences that could easily be aligned. Details on these strains are given in materials and methods. A total of six different haplotypes differ from each other by small insertions and deletions as well as single amino-acid changes. Strains sim2 and sim3 had the same sequence as strain Oxnard; strains maz1 and C167.4 had the same sequence as maz6. B) Comparison of *D. simulans* (strain maz6 is shown) and *D. sechellia* (two strains analyzed) sequences shows that they are composed of highly related stretches (indicated in red, green, and yellow) whose overall arrangement differs. In *D. simulans*, there is an imperfect, tandem repeat of the red + green + yellow stretches (118 or 121 aa long). In *D. sechellia*, a single copy of the red stretch is followed by three repeats (15 aa long) of the green stretch, and then one copy of the yellow stretch.
(PDF)

Figure S3 **Ago2 from** ***Drosophila melanogaster*** **,** ***simulans*** **and** ***sechellia*** **are dramatically variable in the NTD, but highly similar in the rest of the protein.** Ago2 from *D. melanogaster*, *simulans* and *sechellia* aligned with CLUSTALW. Color code for domains as in Fig. S4.
(PDF)

Figure S4 **Sequences of Ago2 family members in insects and in** ***Daphnia pulex*** For various species, Ago2 family members were identified by searching FlyBase annotations or GenBank entries. In a few cases, predicted proteins were corrected based on cDNA data or after resequencing problematic genomic regions; if alternative predictions for the protein included a glutamine- rich NTD, those predictions are listed below.In these sequences, four domains were identified: NTD (light blue), DUF1785 (pink), PAZ (green) and Piwi (yellow). The latter three domains were estimated using the Conserved Domain Search at NCBI. The extent of the NTD was based on comparison to human Ago2 (see Materials and Methods). Glutamine residues are highlighted in red and bold. For *D. pseudoobscura*, *D. persimilis*, and *D. willistoni*, FlyBase annotations suggest the existence of multiple Ago2 paralogs. Sequences of these putative paralogs were compared to ensure that they did not represent alternative isoforms of the same gene. For annotations that showed only minor differences from each other, only one such annotation was included in the list below. The phylogram in Fig. 7 suggests that the thus chosen candidates represent *bona fide* paralogs. **Dsim\Ago2** *Drosophila simulans* NTD sequence based on our cDNA sequencing; rest of sequence based on Genbank entry EDX10559.1. The FlyBase annotation for this gene (GD14553) is largely identical across the NTD, DUF and PAZ domains, but predicts a deletion of much of the Piwi domain. **Dsec\Ago2** *Drosophila sechellia* NTD sequence based on our cDNA sequencing; rest of sequence based on combining FlyBase annotations GM25537 and GM25538 and resequencing the genomic DNA between those annotations. **Dere\Ago2** *Drosophila erecta* Based on alternative GNOMON prediction for FlyBase annotation GG15907. **Dyak\Ago2** *Drosophila yakuba* FlyBase annotation GE22249. **Dana\Ago2** *Drosophila ananassae* FlyBase annotation GF10056. **Dpse\Ago2a** *Drosophila pseudoobscura* FlyBase annotation GA28114. Note: because this prediction does not start with a Methionine, future analysis will likely refine the translation start site. **Dpse\Ago2b** *Drosophila pseudoobscura* FlyBase annotation GA27454. **Dpse\Ago2c** *Drosophila pseudoobscura* FlyBase annotation GA27411. **Dpse\Ago2d** *Drosophila pseudoobscura* Based on alternative GNOMON prediction for FlyBase annotation GA23629. **Dpse\Ago2e** *Drosophila pseudoobscura* FlyBase annotation GA26008. **Dper\Ago2a** *Drosophila persimilis* FlyBase annotation GL14308. **Dper\Ago2b** *Drosophila persimilis* FlyBase annotation GL21510. **Dper\Ago2c** *Drosophila persimilis* Based on alternative N-SCAN prediction for FlyBase annotation GL22202. **Dper\Ago2d** *Drosophila persimilis* FlyBase annotation GL24877. **Dper\Ago2e** *Drosophila persimilis* Flybase annotation GL19556. **Dwil\Ago2a** *Drosophila willistoni* FlyBase annotation GK10600. **Dwil\Ago2b** *Drosophila willistoni* FlyBase annotation GK24525. **Dmoj\Ago2** *Drosophila mojavensis* FlyBase annotation GI13119. **Dgri\Ago2** *Drosophila grimshawi* FlyBase annotation GH14741. **Dvir\Ago2** *Drosophila virilis* Based on alternative N-SCAN prediction for FlyBase annotation GJ17143. **Cqu\Ago2-1** *Culex quinquefasciatus* (Southern house mosquito) GenBank entry XP_001865113. **Cqu\Ago2-2** *Culex quinquefasciatus* (Southern house mosquito) GenBank entry EDS33578.1. **Aae\Ago2** *Aedes aegypti* (yellow fewer mosquito) GenBank entry ACR56327.1. **Amel\Ago2** *Apis mellifera* (honey bee) GenBank entry XP_395048. **Nvit\Ago2** *Nasonia vitripennis* (jewel wasp) GenBank entry XP_001607164.1. **Bmor\Ago2** *Bombyx mori* (domestic silkworm) GenBank entry NP_001036995. **Tcas\Ago2a** *Tribolium castaneum* (red flour beetle) GenBank entry NP_001107842 (Argonaute-2a). **Tcas\Ago2b** *Tribolium castaneum* (red flour beetle) GenBank entry NP_001107828 (Argonaute-2b). **Apis\Ago2** *Acyrthosiphon pisum* (pea aphid) GenBank entry XP_001944852.1. **Phum\Ago2** *Pediculus humanus* (human louse) GenBank entry EEB09910. **Dpul\Ago2** *Daphnia pulex* (common water flea) One of two alternative predictions on WfleaBase: DP_DGIL_SNO_00003046. **Hsap\Ago2** *Homo sapiens* GenBank entry NP_036286.2.
(DOC)

Figure S5 **Comparison of Dpse\Ago2c and Dper\Ago2c.** Dpse\Ago2c and Dper\Ago2c were aligned by CLUSTALW. The two proteins are almost completely identical through the entire coding region. The major differences are in the center of the NTD, where alignment is spotty, suggesting rapid changes in primary sequence. Color code for domains as in Fig. S4.
(DOC)

Table S1 **Evidence for expression of Ago2 NTDs in various insect species.** For various insect Ago2s, the genomic regions corresponding to the NTD are indeed transcribed. Evidence includes cDNA analysis of Ago2 specifically, EST data from high-throughput sequencing efforts, and RNAseq data. If the transcribed region encompasses the entire NTD, this is indicated as “full” in the table.
(DOC)
