# Supplementary material for: Natural Variation of the Amino-Terminal Glutamine-Rich Domain in Drosophila Argonaute2 Is Not Associated with Developmental Defects
Source: PLoS One. 2010 Dec 17;5(12):e15264. doi: 10.1371/journal.pone.0015264 (PMC3002974; doi:10.1371/journal.pone.0015264)
Supplement: Figure S1 — Amino acid sequence of GRR2 repeats in various D. melanogaster strains. GRR2 repeats display slight sequence variations, indicated by different colors. Fly strains differ both in number and type of repeat. The 3CPA122 pattern is identical to the one in the current FlyBase annotation for Ago2, and was also found in strains In(1)AB, w-14 melbourne, red e, and dop46. Note that the same overall repeat copy number can be achieved via distinct primary sequences (compare left and right columns below). This variability in sequence likely results in even greater number of distinct haplotypes, and the ten haplotypes in Table 1, which are based solely on repeat copy number, probably underestimate the true variability among the 32 strains surveyed. (PDF) [file pone.0015264.s001.pdf]

Figure S1:

dop^{1}

QGGH**Q**QGRQGQ**D**GGYQQRP**P**GQQ
QGGH**Q**QGRQGQ**E**GGYQQRP**P**GQQ
QGGH**Q**QGRQGQ**E**GGYQQRP**S**GQQ
QGGH**Q**QGRQGQ**E**GGYQQRP**P**GQQ
QGGH**Q**QGRQGQ**E**GGYQQRP**S**GQQ
QGGH**Q**QGRQGQ**E**GGYQQRP**P**GQQ
QGGH**Q**QGRQGQ**E**GGYQQRP**P**GQQ
QGGH**E**QGRQGQ**E**GGYQQRP**S**GQQ
QGGH**Q**QGRQGQ**E**GGYQQRP**S**GQQ
QGGH**Q**QGRQGQ**E**GGYQQRP**P**GQQ

3CPA103

QGGH**Q**QGRQGQ**D**GGYQQRP**P**GQQ
QGGH**Q**QGRQGQ**E**GGYQQRP**P**GQQ
QGGH**Q**QGRQGQ**E**GGYQQRP**S**GQQ
QGGH**Q**QGRQGQ**E**GGYQQRP**P**GQQ
QGGH**Q**QGRQGQ**E**GGYQQRP**P**GQQ
QGGH**E**QGRQGQ**E**GGYQQRP**P**GQQ
QGGH**Q**QGRQGQ**E**GGYQQRP**S**GQQ
QGGH**Q**QGRQGQ**E**GGYQQRP**H**GQQ
QGGH**Q**QGRQGQ**E**GGYQQRP**P**GQQ
QGGH**Q**QGRQGQ**E**GGYQQRP**P**GQQ

3CPA122

QGGH**Q**QGRQGQ**D**GGYQQRP**P**GQQ
QGGH**Q**QGRQGQ**E**GGYQQRP**P**GQQ
QGGH**Q**QGRQGQ**E**GGYQQRP**S**GQQ
QGGH**Q**QGRQGQ**E**GGYQQRP**P**GQQ
QGGH**Q**QGRQGQ**E**GGYQQRP**S**GQQ
QGGH**Q**QGRQGQ**E**GGYQQRP**P**GQQ
QGGH**Q**QGRQGQ**E**GGYQQRP**P**GQQ
QGGH**E**QGRQGQ**E**GGYQQRP**S**GQQ
QGGH**Q**QGRQGQ**E**GGYQQRP**S**GQQ
QGGH**Q**QGRQGQ**E**GGYQQRP**S**GQQ
QGGH**Q**QGRQGQ**E**GGYQQRP**P**GQQ

Tai255.1

QGGH**Q**QGRQGQ**D**GGYQQRP**P**GQQ
QGGH**Q**QGRQGQ**E**GGYQQRP**P**GQQ
QGGH**Q**QGRQGQ**E**GGYQQRP**S**GQQ
QGGH**Q**QGRQGQ**E**GGYQQRP**P**GQQ
QGGH**Q**QGRQGQ**E**GGYQQRP**P**GQQ
QGGH**Q**QGRQGQ**E**GGYQQRP**P**GQQ
QGGH**E**QGRQGQ**E**GGYQQRP**P**GQQ
QGGH**Q**QGRQGQ**E**GGYQQRP**S**GQQ
QGGH**Q**QGRQGQ**E**GGYQQRP**P**GQQ
QGGH**Q**QGRQGQ**E**GGYQQRP**P**GQQ
QGGH**Q**QGRQGQ**E**GGYQQRP**P**GQQ

TM6B

QGGH**Q**QGRQGQ**D**GGYQQRP**P**GQQ
QGGH**Q**QGRQGQ**E**GGYQQRP**P**GQQ
QGGH**Q**QGRQGQ**E**GGYQQRP**S**GQQ
QGGH**Q**QGRQGQ**E**GGYQQRP**P**GQQ
QGGH**Q**QGRQGQ**E**GGYQQRP**P**GQQ
QGGH**Q**QGRQGQ**E**GGYQQRP**P**GQQ
QGGH**Q**QGRQGQ**E**GGYQQRP**P**GQQ
QGGH**E**QGRQGQ**E**GGYQQRP**P**GQQ
QGGH**Q**QGRQGQ**E**GGYQQRP**S**GQQ
QGGH**Q**QGRQGQ**E**GGYQQRP**P**GQQ
QGGH**Q**QGRQGQ**E**GGYQQRP**P**GQQ
QGGH**Q**QGRQGQ**E**GGYQQRP**P**GQQ

PA120

QGGH**Q**QGRQGQ**D**GGYQQRP**P**GQQ
QGGH**Q**QGRQGQ**E**GGYQQRP**S**GQQ
QGGH**Q**QGRQGQ**E**GGYQQRP**P**GQQ
QGGH**Q**QGRQGQ**E**GGYQQRP**P**GQQ
QGGH**E**QGRQGQ**E**GGYQQRP**P**GQQ
QGGH**Q**QGRQGQ**E**GGYQQRP**S**GQQ
QGGH**Q**QGRQGQ**E**GGYQQRP**P**GQQ
QGGH**Q**QGRQGQ**E**GGYQQRP**P**GQQ
QGGH**E**QGRQGQ**E**GGYQQRP**P**GQQ
QGGH**Q**QGRQGQ**E**GGYQQRP**P**GQQ
QGGH**Q**QGRQGQ**E**GGYQQRP**H**GQQ
QGGH**Q**QGRQGQ**E**GGYQQRP**P**GQQ
QGGH**Q**QGRQGQ**E**GGYQQRP**P**GQQ

*OreR**

QGGH**Q**QGRQGQ**D**GGYQQRP**P**GQQ
QGGH**Q**QGRQGQ**E**GGYQQRP**P**GQQ
QGGH**Q**QGRQGQ**D**GGYQQRP**P**GQQ
QGGH**Q**QGRQGQ**E**GGYQQRP**P**GQQ
QGGH**Q**QGRQGQ**E**GGYQQRP**S**GQQ
QGGH**Q**QGRQGQ**E**GGYQQRP**P**GQQ
QGGH**Q**QGRQGQ**E**GGYQQRP**S**GQQ
QGGH**Q**QGRQGQ**E**GGYQQRP**P**GQQ
QGGH**Q**QGRQGQ**E**GGYQQRP**P**GQQ
QGGH**E**QGRQGQ**E**GGYQQRP**S**GQQ
QGGH**Q**QGRQGQ**E**GGYQQRP**S**GQQ
QGGH**Q**QGRQGQ**E**GGYQQRP**S**GQQ
QGGH**Q**QGRQGQ**E**GGYQQRP**P**GQQ

*OreR***

QGGH**Q**QGRQGQ**D**GGYQQRP**P**GQQ
QGGH**Q**QGRQGQ**E**GGYQQRP**P**GQQ
QGGH**Q**QGRQGQ**E**GGYQQRP**S**GQQ
QGGH**Q**QGRQGQ**E**GGYQQRP**P**GQQ
QGGH**Q**QGRQGQ**E**GGYQQRP**S**GQQ
QGGH**Q**QGRQGQ**E**GGYQQRP**P**GQQ
QGGH**Q**QGRQGQ**E**GGYQQRP**P**GQQ
QGGH**E**QGRQGQ**E**GGYQQRP**S**GQQ
QGGH**Q**QGRQGQ**E**GGYQQRP**P**GQQ
QGGH**Q**QGRQGQ**E**GGYQQRP**S**GQQ
QGGH**Q**QGRQGQ**E**GGYQQRP**P**GQQ
QGGH**Q**QGRQGQ**E**GGYQQRP**P**GQQ
QGGH**E**QGRQGQ**E**GGYQQRP**S**GQQ
QGGH**Q**QGRQGQ**E**GGYQQRP**S**GQQ
QGGH**Q**QGRQGQ**E**GGYQQRP**S**GQQ
QGGH**Q**QGRQGQ**E**GGYQQRP**P**GQQ
