# Supplementary material for: Natural Variation of the Amino-Terminal Glutamine-Rich Domain in Drosophila Argonaute2 Is Not Associated with Developmental Defects
Source: PLoS One. 2010 Dec 17;5(12):e15264. doi: 10.1371/journal.pone.0015264 (PMC3002974; doi:10.1371/journal.pone.0015264)
Supplement: Figure S2 — Variability in the Ago2 NTD for Drosophila simulans , mauritiana , and sechellia strains. The genomic DNA corresponding to exon 3 of ago2 was sequenced in a number of strains of the D. simulans species complex. The amino acid sequences for the corresponding NTD stretches were compared. A) Nine D. simulans and one D. mauritiana strain displayed highly similar sequences that could easily be aligned. Details on these strains are given in materials and methods. A total of six different haplotypes differ from each other by small insertions and deletions as well as single amino-acid changes. Strains sim2 and sim3 had the same sequence as strain Oxnard; strains maz1 and C167.4 had the same sequence as maz6. B) Comparison of D. simulans (strain maz6 is shown) and D. sechellia (two strains analyzed) sequences shows that they are composed of highly related stretches (indicated in red, green, and yellow) whose overall arrangement differs. In D. simulans, there is an imperfect, tandem repeat of the red + green + yellow stretches (118 or 121 aa long). In D. sechellia, a single copy of the red stretch is followed by three repeats (15 aa long) of the green stretch, and then one copy of the yellow stretch. (PDF) [file pone.0015264.s002.pdf]

Figure S2:

A

```
sim1             GLGQQPSTSSGGGQKQKFQGWAGQKTQGQARDGSVDQQQGQWRPAQGQQRGQQQGRQGQE 60
Oxnard           GLEQQPSTSSGGGQKQKFQGWTGQKTQGQARDGSGYQQQGQWRPAQGQQRGQQ---QGQE 57
vermilion        GLEQQPSTSSGGGQKQKFQGWAGQKTQGQARDGSGYQQQGQWRPEQGQQRGQQQGRQGQE 60
maz6             GLEQQPSTSSGGGQKQKFQGWTGQKTQGQARDGSGYQQQGQWRPAQGQQRGQQQGRQGQE 60
D. mauritiana    GLEQQPSTSSGGGQKQKFQGWTGQKTQGQARDGSGYQQQGQWRPAQGQQRGQQQGRQGQE 60
Tsimbazaza       GLEQQPSTSSGGGQKQKFQGWTGQTTQGQARDGSGYQQQGQWRPAQGQQRGQQQGRQGQE 60
                 ** ******************:**.*********  ******** ********   ****

sim1             GGYQQRPPAQQQGGHQQGPQGWSAQGQKGGYQQGGQRQYGGYQQGQGGYQTQSQGQYQSR 120
Oxnard           GGYQQRPPAQQQGGHQQGPQGWPAQGQKGGYQQGGQRQYGGYQQGQGGYQTQSQGQYQSR 117
vermilion        GGYQQRPPAQQQGGHQQGPQGWPAQGQKGGYQQGGQRQYGGYQQGQGGYQTQSQGQYQSR 120
maz6             GGYQQRPPAQQQGGHQQGPQGWPAQGQKGGYQQGGQRKDGGYQQGQGGYQTQSQGQYQSR 120
D. mauritiana    GGYQQRPPAQQQGGHQQGPQGWPAQGQKGGYQQGGQRKDGGYQQGQGGYQTQSQGQYQSR 120
Tsimbazaza       GGYQQRPPAQQQGGHQQGPQGWPAQGQKGGYQQG-----------QGGYQTQSQGQYQSR 109
                 **********************.***********           ***************

sim1             GPPQQQPSTSSGGGQKQKFQGWAGQKTQGQARDGSVDQQQGQWRPAQGPQRGQQ---QGQ 177
Oxnard           GPPQQQPSTSSGGGQKQKFQGWAGQKTQGQARDGSVDQQQGQWRPAQGPQRGQQ---QGQ 174
vermilion        GPPQQQPSTSSGGGQKQKFQGWAGQNTQGQARDGSGYQQQGQWRPEQGQQRGQQQGRQGQ 180
maz6             GPPQQQPSTSSGGDQKQKFQGWAGQKTQGQARDGSGDQQQGQWRPAQGPQRGQQ---QGQ 177
D. mauritiana    GPPQQQPSTSSGGDQKQKFQGWAGQKTQGQARDGSGDQQQGQWRPAQGPQRGQQ---QGQ 177
Tsimbazaza       GPPQQQPSTSSGGGQKQKFQGWAGQKTQGQARDGSGDQQQGQWRPAQGPQRGQQQGRQGQ 169
                 *************.***********:*********  ******** ** *****   ***

sim1             EGGYQQRPPAQQQGGHQQGPQGRPAQGQKGGYQQG-----------QGGYQTQSQGQYQS 226
Oxnard           EGGYQQRPPAQQQGGHQQGPQGRPAQGQKGGYQQG-----------QGGYQTQSQGQYQS 223
vermilion        EGGYQQRPPAQQQGGHQQGPQGRPAQGQKGGYQQG-----------QGGYQTQSQGQYQS 229
maz6             EGGYQQRPPAQQQGGHQQGPQGRPAQGQKGGYQQGGQRKDGGYQQGQGGYQTQSQGQHQS 237
D. mauritiana    EGGYQQRPPAQQQGGHQQGPQGRPAQGQKGGYQQGGQRKDGGYQQGQGGYQTQSQGQYQS 237
Tsimbazaza       EGGYQQRPPAQQQGGHQQGPQGRPAQGQKGGYQQG-----------QGGYQTQSQGQYQS 218
                 ***********************************           ***********:**

sim1             RGPPQQQQAAPLPL 240
Oxnard           RGPPQQQQAAPLPL 237
vermilion        RGPPQQQQAAPLPL 243
maz6             RGPPQQQQAAPLPL 251
D. mauritiana    RGPPQQQQAAPLPL 251
Tsimbazaza       RGPPQQQQAAPLPL 232
                 **************
```

B

D. simulans

GLE
QQPSTSSGGGQKQKFQGWTGQKTQGQARDGSGYQQQ
GQWRPAQGQQRGQQQ
GRQGQEGGYQQRPPAQQQGGHQQGPQGWPAQGQKGGYQQGGQRKDGGYQQGQGGYQTQSQGQYQSRGPPQ
QQPSTSSGGDQKQKFQGWAGQKTQGQARDGSGDQQQ
GQWRPAQGPQRGQQQ
---GQEGGYQQRPPAQQQGGHQQGPQGRPAQGQKGGYQQGGQRKDGGYQQGQGGYQTQSQGQHQSRGPPQ
QQQAAPLPL

D. sechellia

GLR
QQPSTSSGGDQKQKFQGWTGQKTQGQARDGSGDQQQ
GQGRPAQGQQRGQQQ
GQGRPAQGQQRGQQQ
GQGRPAQGQQRGQQQ
GRQGQEGGYQQRPPAQQQGGHQQGPQGWPAQGQKGGYQQGGQRQGGGYQQGQGGYQTQSQGQYQSRGPPQ
QQQAAPLPL
