# Supplementary material for: Natural Variation of the Amino-Terminal Glutamine-Rich Domain in Drosophila Argonaute2 Is Not Associated with Developmental Defects
Source: PLoS One. 2010 Dec 17;5(12):e15264. doi: 10.1371/journal.pone.0015264 (PMC3002974; doi:10.1371/journal.pone.0015264)
Supplement: Figure S3 — Ago2 from Drosophila melanogaster , simulans and sechellia are dramatically variable in the NTD, but highly similar in the rest of the protein. Ago2 from D. melanogaster, simulans and sechellia aligned with CLUSTALW. Color code for domains as in Fig. S4. (PDF) [file pone.0015264.s003.pdf]

## Figure S3:

```
Dmel\Ago2        MGKKDKNKKGGQDSAAAPQPQQQQKQQQQRQQQPQQLQQPQQLQQPQQLQQPQQQQQQQP 60
Dsim\Ago2        ---MTVYKKGGQESAAAPEPQQQQPQQ-------------------QQRQQQPLQQQQQ- 37
Dsec\Ago2        MGKKNKYKKGGQEGAAAPEPQEQQEQRQ--------------QQPQQQQQPQQQQQQQ- 45
                        *****:.****:**:** **                   ** **   *****

Dmel\Ago2        HQQQQQSSRQQPSTSSGGSRASGFQQGGQQQKSQDAEGWTAQKKQGKQQVQGWTKQGQQG 120
Dsim\Ago2        ---NQQGLEQQPSTSSGG---------GQKQK---FQGWTGQKTQG-------------- 68
Dsec\Ago2        ---NQQSLRQQPSTSSGG---------DQKQK---FQGWTGQKTQG-------------- 76
                    :**. .*********         .*:**    :***.**.**

Dmel\Ago2        GHQQGRQGQDGGYQQRPPGQQQGGHQQGRQGQEGGYQQRPPGQQQGGHQQGRQGQEGGYQ 180
Dsim\Ago2        ------QARDG----------SGYQQQG--------QWRPAQGQQRGQQ---QGQEGGYQ 101
Dsec\Ago2        ------QARDG----------SGDQQQG--------QGRPAQGQQRGQQ---QGQ----- 104
                       *.:**          .* :***        * **.  ** *:*   ***

Dmel\Ago2        QRPSGQQQGGHQQGRQGQEGGYQQRPPGQQQGGHQQGRQGQEGGYQQRPSGQQQGGHQQG 240
Dsim\Ago2        QRPPAQQQGGHQQGP-------QGWPAQGQKGGYQQGGQRQYGGYQQGQGGYQTQ----- 149
Dsec\Ago2        GRPAQGQQRGQQQG--------QGRPAQGQQRGQQQGRQGQEGGYQQ------------- 143
                  **.  ** *:***         *  *.  *:  * *** * * *****

Dmel\Ago2        RQGQEGGYQQRPPGQQQGGHQQGRQGQEGGYQQRPPGQQQGGHEQGRQGQEGGYQQRPSG 300
Dsim\Ago2        ---SQGQYQSRGPPQQQPSTSSG-----GGQKQKFQGWAGQKTQGQARDGSVDQQQGQWR 201
Dsec\Ago2        ----------RPPAQQQ-----------GGHQQGPQGWP--------------------- 161
                           * * ***             ** :*    *

Dmel\Ago2        QQQGGHQQGRQGQEGGYQQRPSGQQQGGHQQGRQGQEGGYQQRPSGQQQGGHQQGRQGQE 360
Dsim\Ago2        PAQGPQRGQQQGQEGGYQQRPPAQQQGGHQQGPQG-------RPAQGQKGGYQQG----Q 250
Dsec\Ago2        ---------AQGQKGGYQQ-------GGQRQG-----------------GGYQQG----Q 184
                           ***:*****       **::**                 **:***    :

Dmel\Ago2        GGYQQRPPGQQPNQTQSQGQYQSRGPPQQQQAAPLPLPPQPAGSIKRGTIGKPGQVGINY 420
Dsim\Ago2        GGY----------QTQSQGQYQSRGPPQQQQAAPLPLPP-PEGSIKRGTIGRPGQVAINY 299
Dsec\Ago2        GGY----------QTQSQGQYQSRGPPQQQQAAPLPLPPQPAGSIKRGTIGRPGQVAINY 234
                 ***          ************************** * *********:****.***

Dmel\Ago2        LDLDLSKMPSVAYHYDVKIMPERPKKFYRQAFEQFRVDQLGGAVLAYDGKASCYSVDKLP 480
Dsim\Ago2        LDLDMSKMPSVAYHYDVKIMPERPTKFYRQAFEQFRMDQLGGAILAFDGKASCYSVDKLP 359
Dsec\Ago2        LDLDMSKMPSVAYHYDVKIMPDRPKKFYRQAFEQFRMDQLGGAILAFDGKASCYSVDKLP 294
                 ****:****************:**.***********:******:**:*************

Dmel\Ago2        LNSQNPEVTVTDRNGRTLRYTIEIKETGDSTIDLKSLTTYMNDRIFDKPMRAMQCVEVVL 540
Dsim\Ago2        LNTQNPEVTVTDRNGRTLRYTIEIKETADSNIDLKSLTTYMKDRIFDKPMRAMQCLEVVL 419
Dsec\Ago2        LNTQNPEVTVTDRNGRTLRYTIEIKETADSKIDLKSLTTYMKDRIFDKPMRAIQCMEVVL 354
                 **:************************.**.**********:**********:**:****

Dmel\Ago2        ASPCHNKAIRVGRSFFKMSDPNNRHELDDGYEALVGLYQAFMLGDRPFLNVDISHKSFPI 600
Dsim\Ago2        ASPCYKKSIRVGRSFFKMSDPGESYDLKDGYEALVGLYQAFMLGDRPFLNVDISHKSFPM 479
Dsec\Ago2        ASPCYKKSIRVGRSFFKMSDPGESYDLKDGYEALVGLYQAFMLGDRPFLNVDISHKSFPM 414
                 ****::*:*************.: ::*.*******************************:

Dmel\Ago2        SMPMIEYLERFSLKAKINNTTNLDYSRRFLEPFLRGINVVYTPPQSFQSAPRVYRVNGLS 660
Dsim\Ago2        PIPMIQYLEEFSLNGKINNTTNLEYSRRFLEPFLRGINVVYTPPKSFQSAPRVYRVNGLS 539
Dsec\Ago2        PIPMIQYLEEFSLHAKINNTTNLDYSRRFLEPFLKGINVVYTPPKSFQSAPRVYRVNGLS 474
                 .:***:***.***:.********:**********:*********:***************

Dmel\Ago2        RAPASSETFEHDGKKVTIASYFHSRNYPLKFPQLHCLNVGSSIKSILLPIELCSIEEGQA 720
Dsim\Ago2        RAPANSEVFEHDGKKVTIASYFHSRNYPLKYPQLHCLNVGSSVKSVMLPIELCSIEEGQA 599
Dsec\Ago2        RAPANSEIFEHDGKKVTIASYFHSRNYPLKYPQLHCLNVGSSVKSVMLPIELCSIEEGQA 534
                 ****.** *********************:***********:**::*************
```

```
Dmel\Ago2        LNRKDGATQVANMIKYAATSTNVRKRKIMNLLQYFQHNLDPTISRFGIRIANDFIVVSTR 780
Dsim\Ago2        LNRKDGARQVSEMIRFAATSTNVRKGKIMKLMKYFQHNLDPTISRFGIRIANDFIVVSTR 659
Dsec\Ago2        LNRKDGARQVSEMIRFAATSTNVRKGKIMKLMKFFQHNLDPTISRFGIRIANDFIVVSTR 594
                 ******* **::**::********* ***:*:::**************************

Dmel\Ago2        VLSPPQVEYHSKRFTMVKNGSWRMDGMKFLEPKPKAHKCAVLYCDPRSG-RKMNYTQLND 839
Dsim\Ago2        TLNPPQVEYHNKKFSLVNKGSWRMDNMQFLQPKNVAHKWTVLYCDSRSGGHKISYNQIND 719
Dsec\Ago2        ILNPPQVEYHNKKFSLVNKGSWRMDNMQFLQPKNVAHKWTVLYCDSRSGGHKIPYNQIND 654
                  *.*******.*:*::*::******.*:**:**  *** :*****.*** :*: *.*:**

Dmel\Ago2        FGNLIISQGKAVNISLDSDVTYRPFTDDERSLDTIFADLKRSQHDLAIVIIPQFRISYDT 899
Dsim\Ago2        FGRKILSQSKAFNISLDPEVSIRPFTEDERSLDTVFADLKRSHYDLAIVIIPQSRISYDT 779
Dsec\Ago2        FGRKILSQSKAFNISLDSEVSIRPFTDDERSLDTVFADLKRSHYDLAIVIIPQSRISYDT 714
                 **. *:**.**.*****.:*: ****:*******:*******::********* ******

Dmel\Ago2        IKQKAELQHGILTQCIKQFTVERKCNNQTIGNILLKINSKLNGINHKIKDDPRLPMMKNT 959
Dsim\Ago2        IKQKAELQHGILTQCVKQFTVERKCNDQTIGNILLKVNSKLNGINHKIKDDPRLPMLVNT 839
Dsec\Ago2        IKQKAELQHGILTQCVKQFTVERKCNDQTIGNILLKINSKLNGINHKIKDDPRLPMLVNT 774
                 ***************:**********:*********:*******************: **

Dmel\Ago2        MYIGADVTHPSPDQREIPSVVGVAASHDPYGASYNMQYRLQRGALEEIEDMFSITLEHLR 1019
Dsim\Ago2        MYMGADVTHPSPDQREIPSVVGVAASHDPYGASYNMQYRLQRGTLEEIEDMYTVTLEHLR 899
Dsec\Ago2        MYMGADVTHPSPDQREIPSVVGVAASHDPYGASYNMQYRLQRGTLEEIEDMYTVTLEHLR 834
                 **:****************************************:*******:::******

Dmel\Ago2        VYKEYRNAYPDHIIYYRDGVSDGQFPKIKNEELRCIKQACDKVGCKPKICCVIVVKRHHT 1079
Dsim\Ago2        VYKEYRNAYPDHILYYRDGVSDGQFPKIKNEELRHIRQACDKVGCTPKICCVIVVKRHHT 959
Dsec\Ago2        VYKENRKTYPDHILYYRDGVSDGQFPKIKNEELMHLRQACDKVGCKAKICCVIVVKRHHT 894
                 **** *::*****:*******************  ::********..*************

Dmel\Ago2        RFFPSGDVTTSNKFNNVDPGTVVDRTIVHPNEMQFFMVSHQAIQGTAKPTRYNVIENTGN 1139
Dsim\Ago2        RFFPSGVETPSNRFNNVDPGTVVDRTIVHPNEMQFFMVSHQAIQGTAKPTRYNVIENTGN 1019
Dsec\Ago2        RFFPSGVETPSNRFNNVDPGTVVDRTIVHPNEVQFFMVSHQAIQGTAKPTRYNVIENTGN 954
                 ******  *.**:*******************:***************************

Dmel\Ago2        LDIDLLQQLTYNLCHMFPRCNRSVSYPAPAYLAHLVAARGRVYLTGTNRFLDLKKEYAKR 1199
Dsim\Ago2        LDIDLLQQLTYNLCHMFPRCNRSVSYPAPAYLAHLVAARGRVYLTGTHRFLDLKKEYAKR 1079
Dsec\Ago2        LDIDLLQQLTYNLCHMFPRCNRSVSYPAPAYLAHLVAARGRVYLTGTHRFLDLKKEYAKR 1014
                 ***********************************************:************

Dmel\Ago2        TIVPEFMKKNPMYFV 1214
Dsim\Ago2        TIVPEFMKKNPMYFV 1094
Dsec\Ago2        TIVPEFMKKNPMYFV 1029
                 ***************
```
