# Supplementary material for: Natural Variation of the Amino-Terminal Glutamine-Rich Domain in Drosophila Argonaute2 Is Not Associated with Developmental Defects
Source: PLoS One. 2010 Dec 17;5(12):e15264. doi: 10.1371/journal.pone.0015264 (PMC3002974; doi:10.1371/journal.pone.0015264)
Supplement: Figure S4 — Sequences of Ago2 family members in insects and in Daphnia pulex For various species, Ago2 family members were identified by searching FlyBase annotations or GenBank entries. In a few cases, predicted proteins were corrected based on cDNA data or after resequencing problematic genomic regions; if alternative predictions for the protein included a glutamine- rich NTD, those predictions are listed below.In these sequences, four domains were identified: NTD (light blue), DUF1785 (pink), PAZ (green) and Piwi (yellow). The latter three domains were estimated using the Conserved Domain Search at NCBI. The extent of the NTD was based on comparison to human Ago2 (see Materials and Methods). Glutamine residues are highlighted in red and bold. For D. pseudoobscura, D. persimilis, and D. willistoni, FlyBase annotations suggest the existence of multiple Ago2 paralogs. Sequences of these putative paralogs were compared to ensure that they did not represent alternative isoforms of the same gene. For annotations that showed only minor differences from each other, only one such annotation was included in the list below. The phylogram in Fig. 7 suggests that the thus chosen candidates represent bona fide paralogs. Dsim\Ago2 Drosophila simulans NTD sequence based on our cDNA sequencing; rest of sequence based on Genbank entry EDX10559.1. The FlyBase annotation for this gene (GD14553) is largely identical across the NTD, DUF and PAZ domains, but predicts a deletion of much of the Piwi domain. Dsec\Ago2 Drosophila sechellia NTD sequence based on our cDNA sequencing; rest of sequence based on combining FlyBase annotations GM25537 and GM25538 and resequencing the genomic DNA between those annotations. Dere\Ago2 Drosophila erecta Based on alternative GNOMON prediction for FlyBase annotation GG15907. Dyak\Ago2 Drosophila yakuba FlyBase annotation GE22249. Dana\Ago2 Drosophila ananassae FlyBase annotation GF10056. Dpse\Ago2a Drosophila pseudoobscura FlyBase annotation GA28114. Note: because [file pone.0015264.s004.doc]

# Figure S4:

# Dmel\Ago2

MGKKDKNKKGG**Q**DSAAAP**Q**P**QQQQ**K**QQQQ**R**QQQ**P**QQ**L**QQ**P**QQ**L**QQ**P**QQ**L**QQ**P**QQQQQQQ**PH**QQQQQ**SSR**QQ**PSTSSGGSRASGF**QQ**GG**QQQ**KS**Q**DAEGWTA**Q**KK**Q**GK**QQ**V**Q**GWTK**Q**G**QQ**GGH**QQ**GR**Q**G**Q**DGGY**QQ**RPPG**QQQ**GGH**QQ**GR**Q**G**Q**EGGY**QQ**RPPG**QQQ**GGH**QQ**GR**Q**G**Q**EGGY**QQ**RPSG**QQQ**GGH**QQ**GR**Q**G**Q**EGGY**QQ**RPPG**QQQ**GGH**QQ**GR**Q**G**Q**EGGY**QQ**RPSG**QQQ**GGH**QQ**GR**Q**G**Q**EGGY**QQ**RPPG**QQQ**GGH**QQ**GR**Q**G**Q**EGGY**QQ**RPPG**QQQ**GGHE**Q**GR**Q**G**Q**EGGY**QQ**RPSG**QQQ**GGH**QQ**GR**Q**G**Q**EGGY**QQ**RPSG**QQQ**GGH**QQ**GR**Q**G**Q**EGGY**QQ**RPSG**QQQ**GGH**QQ**GR**Q**G**Q**EGGY**QQ**RPPG**QQ**PN**Q**T**Q**S**Q**G**Q**Y**Q**SRGPP**QQQQ**AAPLPL

PP**Q**PAGSIKRGTIGKPG**Q**VGINYLDLDLSKMPSVAYHYDVKIMPERPKKFYR**Q**AFE**Q**FRVD**Q**LGGAVLAYDGKASCYSVDKLPLNS**Q**NPEVTVTDRNGRTLRYTIEIKETGDSTIDLKSLTTYMNDRIFDKPMRAM**Q**CVEVVLASPCHNKAIRVGRSFFKMSDPNNRHELDDGYEALVGLY**Q**AFMLGDRPFLNVDISHKSFPISMPMIEYLERFSLKAKINNTTNLDYSRRFLEPFLRGINVVYTPP**Q**SF**Q**SAPRVYRVNGLSRAPASSETFEHDGKKVTIASYFHSRNYPLKFP**Q**LHCLNVGSSIKSILLPIELCSIEEG**Q**ALNRKDGAT**Q**VANMIKYAATSTNVRKRKIMNLL**Q**YF**Q**HNLDPTISRFGIRIANDFIVVSTRVLSPP**Q**VEYHSKRFTMVKNGSWRMDGMKFLEPKPKAHKCAVLYCDPRSGRKMNYT**Q**LNDFGNLIIS**Q**GKAVNISLDSDVTYRPFTDDERSLDTIFADLKRS**Q**HDLAIVIIP**Q**FRISYDTIK**Q**KAEL**Q**HGILT**Q**CIK**Q**FTVERKCNN**Q**TIGNILLKINSKLNGINHKIKDDPRLPMMKNTMYIGADVTHPSPD**Q**REIPSVVGVAASHDPYGASYNM**Q**YRL**Q**RGALEEIEDMFSITLEHLRVYKEYRNAYPDHIIYYRDGVSDG**Q**FPKIKNEELRCIK**Q**ACDKVGCKPKICCVIVVKRHHTRFFPSGDVTTSNKFNNVDPGTVVDRTIVHPNEM**Q**FFMVSH**Q**AI**Q**GTAKPTRYNVIENTGNLDIDLL**QQ**LTYNLCHMFPRCNRSVSYPAPAYLAHLVAARGRVYLTGTNRFLDLKKEYAKRTIVPEFMKKNPMYFV

# Dsim\Ago2

MTVykkgg**q**esaaapep**qqqq**p**qqqq**r**qqq**pl**qqqqq**n**qq**gle**qq**pstssggg**q**k**q**kf**q**gwtg**q**kt**q**g**q**ardgsgy**qqq**g**q**wrpa**q**g**qq**rg**qqq**g**q**eggy**qq**rppa**qqq**ggh**qq**gp**q**gwpa**q**g**q**kggy**qq**gg**q**r**q**yggy**qq**g**q**ggy**q**t**q**s**q**g**q**y**q**srgpp**qqq**pstssggg**q**k**q**kf**q**gwag**q**kt**q**g**q**ardgsvd**qqq**g**q**wrpa**q**gp**q**rg**qqq**g**q**eggy**qq**rppa**qqq**ggh**qq**gp**q**grpa**q**g**q**kggy**qq**g**q**ggy**q**t**q**s**q**g**q**y**q**srgpp**qqqq**aaplpl

pppegsikrgtigrpg**q**vainyldldmskmpsvayhydvkimperptkfyr**q**afe**q**frmd**q**lggailafdgkascysvdklplnt**q**npevtvtdrngrtlrytieiketadsnidlkslttymkdrifdkpmram**q**clevvlaspcykksirvgrsffkmsdpgesydlkdgyealvgly**q**afmlgdrpflnvdishksfpmpipmi**q**yleefslngkinnttnleysrrflepflrginvvytppksf**q**saprvyrvnglsrapansevfehdgkkvtiasyfhsrnyplkyp**q**lhclnvgssvksvmlpielcsieeg**q**alnrkdgar**q**vsemirfaatstnvrkgkimklmkyf**q**hnldptisrfgiriandfivvstrtlnpp**q**veyhnkkfslvnkgswrmdnm**q**fl**q**pknvahkwtvlycdsrsgghkisyn**q**indfgrkils**q**skafnisldpevsirpftedersldtvfadlkrshydlaiviip**q**srisydtik**q**kael**q**hgilt**q**cvk**q**ftverkcnd**q**tignillkvnsklnginhkikddprlpmlvntmymgadvthpspd**q**reipsvvgvaashdpygasynm**q**yrl**q**rgtleeiedmytvtlehlrvykeyrnaypdhilyyrdgvsdg**q**fpkikneelrhir**q**acdkvgctpkiccvivvkrhhtrffpsgvetpsnrfnnvdpgtvvdrtivhpnem**q**ffmvsh**q**ai**q**gtakptrynvientgnldidll**qq**ltynlchmfprcnrsvsypapaylahlvaargrvyltgthrfldlkkeyakrtivpefmkknpmyfv

# Dsec\Ago2

MGKKNKYKKGG**Q**EGAAAPEP**Q**E**QQ**E**QQ**R**QQQ**P**QQQQQ**P**QQQQQQQ**N**QQ**SLR**QQ**PSTSSGGD**Q**K**Q**KF**Q**GWTG**Q**KT**Q**G**Q**ARDGSGD**QQQ**G**Q**GRPA**Q**G**QQ**RG**QQQ**G**Q**GRPA**Q**G**QQ**RG**QQQ**G**Q**GRPA**Q**G**QQ**RG**QQQ**GR**Q**G**Q**EGGY**QQ**RPPA**QQQ**GGH**QQ**GP**Q**GWPA**Q**G**Q**KGGY**QQ**GG**Q**R**Q**GGGY**QQ**G**Q**GGY**Q**T**Q**S**Q**G**Q**Y**Q**SRGPP**QQQQ**AAPLPL

PP**Q**PAGSIKRGTIGRPG**Q**VAINYLDLDMSKMPSvayhydvkimpdrpkkfyr**q**afe**q**frmd**q**lggailafdgkascysvdklplnt**q**npevtvtdrngrtlrytieiketadskidlkslttymkdrifdkpmrai**q**cmevvlaspcykksirvgrsffkmsdpgesydlkdgyealvgly**q**afmlgdrpflnvdishksfpmpipmi**q**yleefslhakinnttnldysrrflepflkginvvytppksf**q**saprvyrvnglsrapanseifehdgkkvtiasyfhsrnyplkyp**q**lhclnvgssvksvmlpielcsieeg**q**alnrkdgar**q**vsemirfaatstnvrkgkimklmkff**q**hnldptisrfgiriandfivvstrilnpp**q**veyhnkkfslvnkgswrmdnm**q**fl**q**pknvahkwtvlycdsrsgghkipyn**q**indfgrkils**q**skafnisldsevsirpftddersldtvfadlkrshydlaiviip**q**srisydtik**q**kael**q**hgilt**q**cvk**q**ftverkCND**Q**TIGNILLKINSKLNGINHKIKDDPRLPMLVNTMYMGADVTHPSPD**Q**REIPSVVGVAASHDPYGASYNM**Q**YRL**Q**RGTLEEIEDMYTVTLEHLRVYKENRKTYPDHILYYRDGVSDG**Q**FPKIKNEELMHLR**Q**ACDKVGCKAKICCVIVVKRHHTRFFPSGVETPSNRFNNVDPGTVVDRTIVHPNEV**Q**FFMVSH**Q**AI**Q**GTAKPTRYNVIENTGNLDIDLL**QQ**LTYNLCHMFPRCNRSVSYPAPAYLAHLVAARGRVYLTGTHRFLDLKKEYAKRTIVPEFMKKNPMYFV

# Dere\Ago2

MGKNKSW**QQ**SGR**Q**GP**Q**G**QQ**G**Q**G**QQ**GGF**QQ**GP**Q**GR**Q**G**Q**G**QQ**GGF**QQ**GP**Q**GR**Q**G**Q**G**QQ**GGF**QQ**GP**Q**G**QQ**G**Q**G**QQ**GGF**QQ**GP**Q**GR**Q**G**Q**G**QQ**GGF**QQ**GP**Q**GR**Q**G**Q**G**QQ**GGF**QQ**GP**Q**GR**Q**D**Q**G**QQ**GGF**QQ**GP**Q**GR**Q**G**Q**G**QQ**GGF**QQ**GP**Q**GR**Q**G**Q**G**QQ**GGF**QQ**GP**Q**GR**Q**G**Q**G**QQ**GGF**QQ**GP**Q**GR**Q**G**Q**G**QQ**SGS**QQ**GP**Q**G**QQ**G**Q**G**QQ**GGF**QQ**IP**Q**GR**Q**G**Q**G**QQ**SGS**QQ**IP**Q**GR**Q**G**Q**GGGY**QQ**RPPA**QQ**GHG**QQ**GGY**QQ**RPPA**QQ**GHG**QQ**GGY**QQ**HPR**Q**G**Q**GE**Q**GAKS**Q**G**Q**Y**Q**SRGPP**QQQQ**AAPL

PP**Q**PAGSIKRGTIGKPG**Q**VAVNYLDIDMSKMPPVAYHYDVKIMPERPKKFYR**Q**AFE**Q**FRMN**Q**LGGAIAAYDGRASCYSVDKLPLKS**Q**NSEVTVTDRNGRTLHYTIEIKETNDSNIDLKSLTTYMKDRIFDKPMRAM**Q**CMEVVLASPCHNKAIRAGRSFFKMSEPG**Q**RFELDDGYEALVGLY**Q**AFMLGDKPFLNVDISHKSFPIAMSMIEYLERYGLKARISNTTDLDSSRRFLEPFLKGINVVYTPP**Q**SFASAPRVYKVNGLSRCPASKEIFEHDGKKVTIASYF**Q**SRNYNLKFP**Q**LHCLDAGPPAKRILLPIELCSIEEG**Q**ALNRKDGAT**Q**VANMIKFAATSTNVRKGKIMNMLKFF**Q**HNLDPTISRFGIRIANDFIVVSTRTLNPP**Q**VEY**Q**GNKWCGVRNGSWRMDNMKFLEPKPKAHKWAIVYYDPRHGRKMHFN**Q**VADFER**Q**VLA**Q**SKSVNISLESKAEFRTFMDERGLDDVFADLKRS**Q**HDLAFVIIP**Q**FGTSYDIIK**Q**KAEL**Q**HGILT**Q**CIK**Q**FTVERKLNP**Q**TIGNILLKVNSKLNGINHKIKDDPRLPMLKNTMYMGADVTHPSPD**Q**REIPSVVGVAASHDPYGAAYNM**Q**YRL**Q**RGALEEIEDMYSITLEHLRVYH**Q**YRKTYPEHILYYRDGVSDG**Q**FPKIKNEELRGII**Q**ACSKVGINPKICCVIVVKRHHARFFPNGEPS**Q**YNKFNNVDPGTVVDRTIVHPNEM**Q**FFMVSH**Q**SI**Q**GTAKPTRYNVIENTGNLDIDLL**QQ**LTYNLCHMFPRCNRSVSYPAPAYLAHLVAARGRVYLTGSTRFLDLKKEYAKRTIVPEFMKTNPMYFV

# Dyak\Ago2

MGKKNKFKAEGKPSDATAPT**QQQQQQ**P**QQQQQQQ**P**QQQQQQ**P**QQQQQQQQQQ**ALG**QQ**AAR**QQ**PTSSGW**Q**G**Q**G**Q**R**Q**GA**Q**G**Q**G**QQ**GGF**QQ**RPPA**QQ**G**Q**G**QQ**GGY**QQ**RPPAN**Q**G**Q**G**QQ**GGS**QQ**RPPA**QQ**G**Q**G**QQ**GGY**QQ**RPPAN**Q**G**Q**G**QQ**GGY**QQ**RPPG**QQ**G**Q**G**QQ**GGF**QQ**RPPA**QQ**G**Q**G**QQ**GGY**QQ**RPPAN**Q**G**Q**G**QQ**GGY**QQ**RPPA**QQ**G**Q**G**QQ**GGY**QQ**RPPAN**Q**G**Q**G**QQ**GGY**QQ**RPPG**QQ**G**Q**G**QQ**GGF**QQ**RPPA**QQ**G**Q**G**QQ**GGY**QQ**RPSAN**Q**G**Q**G**QQ**GGY**QQ**RPPA**QQ**G**Q**G**QQ**GGY**QQ**RPPA**QQ**G**Q**G**QQ**GGY**QQ**RPPG**QQ**G**Q**G**QQ**GGY**QQ**RPPA**QQ**HGA**Q**S**Q**G**Q**Y**Q**SRGPP**QQQQ**AVAL

PP**Q**PAGSIKRGTIGKPG**Q**VGVNYLDIDMSKMPPVAYHYDVRIMPERPKKFYRHAFE**Q**FRMN**Q**LGGAIVAFDGRASCYSVDKLPVKS**Q**NPEVTVTDRNGRTLRYTIEIKETNDPSIDLNSLTTYMKDRIFEKPMRAM**Q**CLEVVLASPCHNKAIRAGRSFFKMSEPG**Q**RFELDDGYEALVGLY**Q**AFMLGDKPFLNVDISHKSFPIAMSMIEYLELYGIKAKINN**Q**TNL**Q**NSRRFLEPFLRGINVVYTPP**Q**SFASAPRVYKVNGLSSGPASSETFESDGKKVTIAAYF**Q**SRNYNLKFP**Q**LHCLHVGPPTKHILLPIELCTIEEG**Q**ALNRKDGAT**Q**VANMIKFAATSTNVRKNKIMNLLKFFEHNLDPTISRFGIRIANDFIMVSTRTLNPP**Q**VEY**Q**GNRYCGVRNGSWRMDNMKFLEPKPKAHKWAILYFDPKYGRKIHFN**Q**VADFERNVLG**Q**SKSVNISLESKAEIRTFSDDRSLDDVFADLKRS**Q**HDLAFVIIP**Q**SGSSYDIIK**Q**KAEL**Q**HGILT**Q**CIK**Q**YTFDRKLNP**Q**TIGNILLKVNSKLNGINHKIKDDPRLPMLKNAMYMGADVTHPSPD**Q**REIPSVVGVAASHDPYGAAYNM**Q**YRL**Q**RGALEEIEDMYAITLEHLRVYH**Q**YRKAYPEHILYYRDGVSDG**Q**FPKIKNEELRGIN**Q**ACAKVGIKPKLCCVIVVKRHHTRFFPNGEPS**Q**YNKFNNVDPGTVVDRTIVHPNEM**Q**FFMVSH**Q**SI**Q**GTAKPTRYNVIENTGNLDIDLL**QQ**LTYNLCHMFPRCNRSVSYPAPAYLAHLVAARGRVYLTGSTRFLDLKKEYAKRTIVPEFMKTNPMYFV

# Dana\Ago2

mgkknkykddgkp**qqqqqqqqq**p**qqqqqq**p**q**assgapvg**q**p**q**r**qq**gggg**q**ga**qq**rnpl**qq**lep**q**vgptps**q**g**qq**g**q**ggwsrvps**qq**pgrs**qqqq**shg**q**gg**qqqqqq**shgkgg**qq**r**q**py**q**g**q**gg**qq**r**q**py**q**g**q**gg**qq**r**q**py**q**g**q**gg**qq**ry**qq**gssgf**q**ggn**qq**rp**qq**g**q**pgt**qq**rp**qq**gpgps**q**ggyr**qq**gshp**q**p**q**rs**q**ggavapslpagsikr

gtigrpgevavnyldinmekmpatayhydvkimperpkkfyr**q**afe**q**yrvn**q**lggaiaaydgkascysvdklktns**q**npevtvtdlhgrtlrytveiketgdtevnlnslksymterifdkpmram**q**clevvlaspchnkairagrsffkmsepg**q**rreledgyealvgly**q**afmlgdkpflnvdishksfpiampvleylerfglk**q**rinastsld**q**srrfiepflkginivytpptsfgtasrvykvnglsaypsnk**q**tfvlegktltvsdyfksrnyvlkypsl**q**clhvgppvkniyvpielchieag**q**alnrkdgat**q**vanmikfaatstnvrkekimhlldffkhnldptisrfgiriandfivvhtrtlnap**q**leykdnkwasprngswrmdnmkflepknkahkwavlycnggrpipfs**q**lsdfer**q**mln**q**sksvnvvleakadirpfkddrdld**q**cfvdlkkn**q**cdlafviipnygasyetik**q**kaelkhgilt**q**cik**q**ftferklnp**q**tignillkvnsklnginhklkedtrlpvpknamflgadvthpspd**q**reipsvvgvaashdpygaaynm**q**yrl**q**raaleeiedmesitlahlsvykkfrgkypehiiyyrdgvsdg**q**fpkikneelrgikaacakvainpkiccvivvkrhhtrffpkgeps**q**ynkfnnvdpgtvvdrtivhpnem**q**ffmvsh**q**si**q**gtakptrynvientgnldidvi**qq**mtynlchmfprcnrsvsypapaylahlvaargrvylngsnrfepnlkkeyekrkivevflmtnpmyfv

# Dpse\Ago2a

ssslheslaagasadgeeathppnggpaasgl**q**keeek**q**aewkt**q**rs**q**krrykagegwggkkr**qqq**phsdasekhnr**qq**hag**qq**rg**q**ggn**qq**c**q**ggs**q**lyssr**q**p**q**gelp**q**wpg**q**prgtlgkpg**q**athppnggpaasgl**q**keeek**q**aewkt**q**rs**q**krrykagegwggkkr**qqqqq**phsdasekhnr**qq**hag**qq**rg**q**ggn**qq**c**q**ggs**q**lyssr**q**p**q**gelp**q**wpg**q**pr

gtlgkpg**q**vsvnyldvnldkmpavayhydvkitperpkkfyr**q**afe**q**yrvehlggaiaafdgrascysvvklkcss**q**g**q**evkvtdrhgrtlnytlelketedlevdlnslrsymkdkiydkpmral**q**clevvlaapchntairagrsffkrsepgsafdlndgyealvgly**q**tfvlgdrpfvnvdishksfpkamtiidylely**q**k**q**kidkstnldykrsdiesfltgmniiyeppaclgsaprvfrvnglckvpast**q**tfeldgkemtvaeyyksr**q**ynlkfpnllclhvgpplkhiylpielcriedg**q**tlnrkdgan**q**vaamikyaatstnerkakiirlmeyfrhnldptishfgirlgsdfivvntrtlnap**q**ieyknslasvrngswrmdgm**q**flepkpkshkwailygkinylyvdel**q**kmvi**q**ksrkvnlcldakaeklyykdereldalfryfkkn**q**fdvvfviipnsghlydvvk**q**kael**q**hgilt**q**cik**q**itverkcna**q**vignillkvnsklngtnhklrddlhclpkktmflgadvthpspd**q**reipsvvgvaashdpfgasynm**q**yrl**q**rsaleeiedmesitlehlrvyhnf**qq**cypdhiiyyrdgvsdg**q**fpniknkelrgisaacsklhikpkiccfivvkrhhtrffpngvps**q**ynkfnnvvtgtvvdrtivhpnem**q**ffmvsh**q**si**q**gtakptrynvientgnldidvl**q**kltynlchmfprcnravsypapaylahlaaargrvyltgctkfrsp**q**eeyakrlilpefmktnpmyfv

# Dpse\Ago2b

mgkenkykpnaekpskptikryfgkpl**q**eh**q**lkatgasaegdkvthppyggpaasgl**q**k**q**k**q**eee**q**gew**q**t**q**rc**q**krrdkagegwggk**qqqqqqqq**lp**qq**rg**q**hrdpiek**q**n**qqqq**tv**q**rrl**q**ghne**q**h**q**ggs**q**fygppkp**q**delpkwpra**q**nrglpl**q**pl**q**nllp**q**t**q**kvvpplpagtmkr

gtlgkpg**q**vsvnylevnldrmpavay**q**ydvkitsvcpkkfyr**q**afe**q**yrvehlggaiaaydgrgscysvvklkcsp**q**g**q**emkvtdrhgrtlnytvelrkt**q**esevdlsslrsymkdkiydkpmral**q**clevvlaapchntarragrsffkgsdpgntfdlkdgyealvgly**q**tfvlgdrpfvnvdishkafpkamsiidyie**q**y**q**r**q**kidkstnldyrrsdiesflndiniiydppacfgsaprvfrvnglskapast**q**tfeldgkettvakyfksreydlkfpnllclhvgpplkhiylpielcriddg**q**tmkrkdtaarvaailkfaatstnerkakivrlleyfkhnldptishfgirlgtdfivvntrtlnap**q**ieyknnnlasvrngswrmdrm**q**ffepkpkphkwailygkiny**q**yvdel**q**kmvl**qq**srtvnlcldtkadkrnykdereldahfhdfkrn**q**fdlvfviipnvgrsydvvk**q**kaelkhgilt**q**clk**q**itverkcnp**q**cignvllkvnsklnginhklrddprcllknamflgadvthpspd**q**wempsvvgvaashdpfgasynm**q**yrl**q**rstleeiedmesitlehlrvyy**q**frksypehiiyycdgfgdc**q**ypkikseelrgitaacckmhikpkiccvivvkrhhtrffpsgaps**q**yn**q**lnnvdpgtvvdrtivhpnemeffmvsh**q**an**q**gtakptrysvientgnldidvl**qq**ltfnlchmfprcnravsypapaylahlaaargrvyltgstmfrsp**q**eeyakrlivpdfmktnpmyfv

# Dpse\Ago2c

mgkknkykeaekpvppaa**q**p**qqqqq**aaaapga**q**nrpt**q**tpstssaasgs**qqqq**ggwrt**q**dsh**qq**rs**q**ag**q**gw**qqq**ggg**qq**rgp**qqq**ggg**qq**rgpp**qqq**ggy**qq**rp**q**g**qq**a**q**g**q**yrgpp**qqq**ggy**qq**rp**q**g**qq**a**q**g**q**yrgpp**qqq**ggy**qq**rp**q**s**qq**gr

v**q**ggaalpplpagtmkrgtlgkpg**q**vsvnyldvnldkmpavayhydvkitperpkkfyr**q**afd**q**yrvehlggaiaafdgrasaysavklkcss**q**g**q**evkildrhgrtltytvelketedlevdlnslrnymknkiydkpmral**q**clevvlaapchntairagrsffkrsepgkafdlndgyealvgly**q**tfvlgdrpfvnvdishksfpkampiieyie**q**y**q**r**q**kidkstnldyrrydiesflkgmniiydppaclasaprvfrvngltkfpasslkfeldgk**q**ttvadyfrsrkynlmypnllclhvgpplkniylpielcriedg**q**alnrkdgan**q**vaamikyaatstnerkakiihlleyfkhnldptishfgirlendfivvhtrtlnap**q**veyknnnlasvrngswrmdrm**q**ffepkpkphkwailhgkinymsvvdf**q**gmii**qq**srtvnvcln**q**kadirnyrdereldshf**q**dfkkn**q**fdlvfviipnsgpfydvvk**q**kael**q**hgilt**q**cikeitvlrkcnl**q**cignvllkvnsklnginhklkddprfllknamflgadvthpspd**q**reipsvvgvaashdpfgasynm**q**yrl**q**rsaleeiedmesitlehfrvyh**q**frksypehivyyrdgvsdg**q**fpkikneelrgisaacsklrinpkiccvivvkrhhtrffpngaps**q**ynkfnnvdpgtvvdrtivhpnem**q**ffmvsh**q**si**q**gtakptrynvientgnldidll**qq**ltynlchmfprcnravsypapaylahlaaargrvyltgctkfrtpkeeyakrlivpefmktnpmyfv

# Dpse\Ago2d

MGKKNKYKEAEKPVPPAA**Q**P**QQQ**RAAGAPGA**Q**NRPT**Q**PARPPATPTPSTSSAASGS**Q**L**QQ**GGWRT**Q**DSH**QQ**RS**Q**AG**Q**GWGG**QQ**KGPR**Q**PGGN**QQ**RGP**Q**K**Q**GSG**Q**ERRP**QQQ**GGG**QQ**RGP**QQQ**GGG**QQ**RGPP**Q**K**Q**GGAALPPLPAGTMKR

GTLGKPG**Q**VSVNYLDVNLDKMPAVAYHYDVKITPERPKKFYR**Q**AFE**Q**YRVEHLGGAIAAFDGRASAYSAVKLKCSS**Q**GHEVKILDRHGRTLTYTLEIKETEDSEVDLNSLRNYMKDRIYDKPMRAL**Q**CLEVVLAAPCHNTAiragrsfykrsepgkafdlndgyealvgly**q**afvlgdrpfvnvdishksfpkamtiieyle**q**y**q**rkridkstnlddrrykiesflkgmnivydppacfasaprvfrvnglskfpass**q**kfeldgk**q**ttvaeyfrsrkynlkypnllclhvgpplkniylpielcriedg**q**alnrkdgan**q**vaamikyaatptnerkakiirlmeyfrhnldptishfgirlgsdfivvntrtlnap**q**ieyknklasvwngswrmdgm**q**fydpkpkphkwailygkidyirvvdf**q**gmii**q**lsrtvnvclndnaeirnyldlreldshfldlknn**q**fdlvyviipnsgsvydvvk**q**kaelehgilt**q**cikentvlhkcnl**q**cignvllkvnsklnginhklkddplcllknamflgadvthpspd**q**reipsvvgvaashdpfgasynm**q**yrl**q**rsdleei**q**dmesitlehlrvyh**q**yrksypehivyyrdgvsdg**q**fpkikkeelsgisaactkllinpkiccvivvkrhhtrffpngtpslynkfnnvdpgtvvdrtivhpnem**q**ffmvsh**q**si**q**gtakptrynvientgnldidll**qq**ltynlchmfprcnravsypapaylahlaaargrvyltgctkfltpkeeyekrlivpeflkrnpmyfv

# Dpse\Ago2e

maksknykkkkpvaepgaaaealksd**q**vpeperg**q**gdgprv**q**glkr

gtvgrlgevavnyl**q**vnldrmpavayhydvkfvpelpkkfyrlafdkfrveylggavaafdgrascysveklncrs**q**gaevtvsdpygrklkyavaimetadpevdlnslrtymrdriyekpmral**q**clevvlaapchnkavrsgrsfy**q**lsepgkvhslengeevlfglf**q**alvlgdrpfvnvdithkcfhlampvveylerf**q**tkskita**q**tnlesrrsdidahlkgisvayeppksflsatrvykvnalt**q**ypasr**q**afncdgtkvtvaayfksrghalrfpnllclhvgsp**q**msvylpielcrieek**q**alnrkdskv**q**sagivdiaatstnarkvkilellrhfdynadptisrfgfrlntdfivv**q**trvldppli**q**yrnkasasvrnglwhidrs**q**ffdsrpkahkwailhpglnynkmrdfe**q**fvlshsgrvnmslapkaeirtytdaksldpsfkefkag**q**ydlvlvvipnsgnfydklk**q**kaeleygilt**q**cik**q**atverrcng**q**vvgnlllkmnsklnginhtlkadtaalpknvmfvgadvthpspe**q**reipsvvgvaashdafgasynm**q**yrl**q**rgaleeiedmesiltehlrvyrryr**q**cypehimyyrdgvsdg**q**fpkirneelrgmsvacakigikpkicciivvkrhhtrffpsgcpsesnkfnnvepgtvvdrtivhpnev**q**wfmvsh**q**sikgtarptrynviantgrldidll**qq**mthnlchlfprcnravsypapaylahlaaargrvyltgtttfasp**qq**eykkrlidpalssrnpmyfv

# Dper\Ago2a

mpava

yhydvkitpdrpkkfyr**q**afe**q**yrvehlggaiaafdgrascysvvklkcss**q**g**q**evkvtdrhgrtlnytlelketedlevdlnslrsyvkdkiydkpmral**q**clevvlaapchntairagrsffkrsepgsafdlndgyealvgly**q**tfvlgdrpfvnvdishksfpksmtiidylely**q**kekidkstnldykrsdiesfltgmniiyeppaclgsaprvfrvnglckvpast**q**tfeldgkemtvaeyyksr**q**ynlkfpnllclhvgpplkhiylpielcriedg**q**tlnrkdgan**q**vaamikyaatstnerkakiirlmeyfrhnldptishfgirlgsdfivvntrtlnap**q**ieyknnlasvrngswrmdgm**q**flepkpkshkwailygkinylyvdel**q**kmvi**q**ksrkvnlcldakaeklyykdereldahfryfknn**q**fdvvfviipnfghlydvvk**q**kael**q**hgilt**q**cikrvtverkcna**q**vigsillkvnsklngtnhklrddlhclpkktmflgadvthpspd**q**reipsvvgvaashdpfgasynm**q**yrl**q**rsaleeiedmesitlehlrvyhnf**qq**cypdhiiyyrdgvsdg**q**fpniknkelrgisaacsklhikpkiccfivvkrhhtrffpngvps**q**ynkfnnvvtgtvvdrtivhpnem**q**ffmvsh**q**si**q**gtakptrynvientgnldidvl**q**kltynlchmfprcnravsypapaylahlaaargrvyltgctkfrsp**q**veyakrlivpefmktnpmyfv

# Dper\Ago2b

mgkenkykpnaekptkptieryfgkpl**q**eh**q**vkatgasaegdkvthppyggpaasgl**q**k**q**k**q**eee**q**gew**q**t**q**gc**q**krrdkagegwggk**qqqq**r**qqqq**r**qqqq**r**qqqq**r**qqq**lp**qq**rg**q**hrdpiek**q**n**qqqq**tv**q**rrl**q**ghne**q**h**q**ggs**q**cygppkp**q**delpkwpra**q**nrglpl**q**pl**q**nl**q**nllp**q**t**q**kvvpplpaatmkr

gtlgkpg**q**vsvnylevnldkmpavay**q**ydvkitsvcpkkfyr**q**afe**q**yrvehlggaiaaydgrgscysvvklkcsp**q**g**q**emkvtdrhgrtlnytvelrkt**q**dsevdlsslrsymkdkiydkpmral**q**clevvlaapchntarragrsffkgsdpgntfdlkdgyealvgly**q**tfvlgdrpfvnvdishkafpkamsiidyie**q**y**q**r**q**kidkstnldyrrsdiesflsdiniiydppacfgsaprvfrvnglskapast**q**tfeldgkettvakyfksrkydlkfpnllclhvgpplkhiylpielcriddg**q**tmkrkdtaarvaamlkfaatstnerkakivrlleyfkhnldptishfgirlgtdfivvntrtlnap**q**ieyknnnlasvrngswrmdrm**q**ffepkpkphkwailygkiny**q**yvdel**q**kmvl**qq**srtvnlcldtkadkrnykdereldahfhdfkkn**q**fdlvfviipnvgrsydvvk**q**kaelkygilt**q**clk**q**itverkcnp**q**cignvllkvnsklnginhklrddprcllkntmflgadvthpspd**q**wempsvvgvaashdpfgasynm**q**yrl**q**rstleeiedmesitlehlrvyy**q**frksypehiiyyrdgvshg**q**ypkikskelrgitaacckmhikpkiccvivvkrhhtrffpngaps**q**yn**q**lnnvdpgtvvdrtivhpnemeffmvsh**q**an**q**gtakptrynvientgnldidvl**qq**ltfnlchmfprcnravsypapaylahlaaargrvyltgttmfrsp**q**eeyakrlivpdfmktnpmyfv

# Dper\Ago2c

MGKKNKYKEAEKPVPPAA**Q**P**QQQQQ**GW**QQQ**GGG**QQ**RGP**QQQ**GGG**QQ**RGP**QQQ**GGG**QQ**RGPP**QQQ**GGY**Q**G**QQ**A**Q**G**Q**YRGPP**QQQ**GGY**QQ**RP**Q**G**QQ**A**Q**G**Q**YRGPP**QQQ**GGY**QQ**RP**Q**G**QQ**A**Q**G**Q**YRGPP**QQQ**GGY**QQ**RP**Q**S**QQ**GR

V**Q**GGAALPPLPAGTMKRGTLGKPG**Q**VSVNYLDVNLDKMPAVAYHYDVKITPERPKKFYR**Q**AFD**Q**YRVEHLGGAIAAFDGRASAYSAVKLKCSS**Q**G**Q**EVKILDRHGRTLTYTVELKETEDTEVDLNSLRNYMKNKIYDKPMRAL**Q**CLEVVLAAPCHNTAIRAGRSFFKRSEPGKAYDLNDGYEALVGLY**Q**TFVLGDRPFVNVDISHKSFPKAMTIIEYIE**Q**Y**Q**R**Q**KIDKSTNLDYRRYDIESFLKGMNIIYDPPACLASAPRVFRVNGLTKFPASSLKFELDGK**Q**TTVADYFRSRKYNLMYPNLLCLHVGPPLKNIYLPIELCRIEDG**Q**ALNRKDGAN**Q**VAAMIKYAATSTNERKAKIIHLLEYFKHNLDPTISHFGIRLENDFIVVHTRTLNAP**Q**VEYKNNNLASVRNGSWRMDRM**Q**FFEPKPKPHKWAILHGKINYMSVVDF**Q**GMII**QQ**SRTVNVCLNEKADIRNYRDERELDSHF**Q**DFKKN**Q**FDLVFVIIPNSGPFYDVVK**Q**KAEL**Q**HGILT**Q**CIKEITVLRKCNL**Q**CIGNVLLKVNSKLNGINHKLKDDPRFLLKNAMFLGADVTHPSPD**Q**REIPSVVGVAASHDPFGASYNM**Q**YRL**Q**RSALEEIEDMESITLEHLRVYH**Q**FRKSYPEHIVYYRDGVSDG**Q**FPKIKNEELRGISAACSKMRINPKICCVIVVKRHHTRFFPNGAPS**Q**YNKFNNVDPGTVVDRTIVHPNEM**Q**FFMVSH**Q**SI**Q**GTAKPTRYNVIENTGNLDIDLL**QQ**LTYNLCHMFPRCNRAVSYPAPAYLAHLAAARGRVYLTGCTKFRTPKEEYAKRLIVPEFMKTNPMYFV

# Dper\Ago2d

mlltpsyfikileykeaekpvppaa**q**p**qqqq**aagapga**q**nrpt**q**parppatptpstssaasgs**q**l**qq**ggwrt**q**dsh**qq**rs**q**ag**q**gwdg**q**lkgpr**q**pggs**qq**rgp**q**k**q**gsg**q**errp**qqq**ggg**qq**rgp**qqq**ggg**qq**rgpp**q**k**q**g

gaalpplpagtmkrgtlgkpg**q**vsvnyldvnldkmpavayhydvkitperpkkfyr**q**afd**q**yrvehlggaiaafdgrasaysavklkcss**q**g**q**evkildrhgrtltytleiketedsevdlnslrnymkdriydkpmrvl**q**clevvlaapchntairagrsffkrsepgkafdlndgyealvgly**q**afvlgdrpfvnvdishksfpkamtiieyle**q**y**q**rkridkstnlddrrykiesflkgmnivydppacfasaprvfrvnglskfpass**q**kfeldgk**q**ttvaeyfrsrkynlkypnllclhvgpplkniylpielcriedg**q**alnrkdgan**q**vaamikyaatstnerkakiirlmeyfrhnldptishfgirlgsdfivvntrtlnap**q**ieyknnlasvrngswrmdrm**q**fydpkpkphkwailygkidymsvvdf**q**gmii**q**lsrtvnmclndnaeirnysdeceldshfldlknn**q**fdlvyviipnsgsvydvvk**q**kaelehgilt**q**cikentvlrkcnl**q**cignvllkvnsklnginhklkddplcllknamflgadvthpspd**q**reipsvvgvaashdpfgasynm**q**yrl**q**rsdleei**q**dmesitlehlrvyh**q**frksypehivyyrdgvsdg**q**fpkikkeelsgicaactkmlinpkiccvivvkrhhtrffpngtpslynkfnnvdpgtvvdrtivhpnem**q**ffmvsh**q**si**q**gtakptrynvientgnldidll**qq**ltynlchmfprcnravsypapaylahlaaargrvyltgctkfltpkeeyekrlivp**q**flktnpmyfv

# Dper\Ago2e

maksknykkkkpvaepgaaaevlksd**q**vpeperg**q**gdgprv**q**glkr

gtlgrlgevavnyl**q**vnldrmpavayhydvkfvpelpkkffrlafdkfrvehlggavaafdgrascysveklncrs**q**gaevtvsdpygrklkyavaimetadpevdlnslrtymrdriyekpmral**q**clevvlaapchnkavrsgrsfy**q**lsepgkvhslengeevlfglf**q**alvlgdrpfvnvdithkcfhlampvveylerf**q**mkskita**q**tnlesrrsdidahlkgisvayeppksflsatrvykvnalt**q**ypasr**q**afncdgtkvtvaayfksrghalrfpnllclhvgsp**q**tsvylpielcrieek**q**alnrkdskv**q**sagivdiaatstnarkvkilellrhfdynadptisrfgfrlntdfivv**q**trvldppli**q**yrnkasasvrnglwhidrs**q**ffdsrpkahkwailhpgmnynkmrdfe**q**fvlshsgrvnmslapkaeirtytdaksldpsfkefkag**q**ydlvlvvipnsgnfydklk**q**kaeleygilt**q**cik**q**atverrcng**q**mvgnlllkmnsklnginhtlkadtaalpknvmfvgadvthptpe**q**reipsvvgvaashdafgasynm**q**yrl**q**rgaleeiedmesiltehlrvycryr**q**cypehimyyrdgfsdg**q**fpkimneellgmimacl**q**igikpkicciivvkrhhtrffpsgcpsesnkfnnvepgtvvdrtivhpnev**q**wfmvsh**q**sikgtarptrynvientgrldidll**qq**mthnlchlfprcnravsypapaylahlaaargrvyltgtttfasp**qq**eykkrlidpalssknpmyfv

# Dwil\Ago2a

MGKKNKYK**Q**SEDAPSAPS**QQQQ**AAGGNAPPNP**Q**GTARA**Q**PS**QQ**S**Q**G**Q**GGSSIS**QQ**A**Q**GSGAPS**Q**IS**QQ**S**Q**GGGE**Q**RG**QQ**GGWVG**Q**GGN**QQ**RS**QQ**SAWGG**Q**GGN**QQ**RG**QQ**EGGGAGRSGY**QQ**RG**QQ**GGRGGY**QQ**RG**QQ**EGGGAGRGSY**QQ**RG**QQ**EGGGAGRGGY**QQ**RD**QQ**EGRGGY**QQ**RG**QQ**ESRGGY**QQ**RG**QQ**SRP**Q**GGAAL

PPLPDLPVGSIKRGTIGTPG**Q**VAVNYLNVDMTEMPAKAYHYDVKITPERPKKFFRDAFE**Q**FRIIHLKGAAVAFDGRASCFSVDKLETAGNGGDVKVTDRHGRTLNYNV**Q**IKSTASE**Q**IDLNSLRCYMKDKIYDKPM**Q**AM**Q**CLEVVLAAPCHKKTIRAGRSFFKSSEPN**Q**RLDLGEGYECLIGLY**Q**AFVLGDRPFINVDISHKSFPIALSMLEYLENYGLNSRYATKITTTTNL**QQ**SRTYIE**Q**FIKGINIIYEPPASFNSAPRIFKVNGLSP**Q**SAD**QQ**KF**Q**LEDKKETTVKEYFRGRNYILKYPNLHCLHVGPPAK**Q**IYVPIELCRIEEG**Q**ALNRKDGTN**Q**VSAMIKYAATSTNERKGKIINLLKYI**Q**HN**Q**DPTISRFGIKIVGDFITVHTRTLNPP**Q**VEYKNKFMTSVRNGSWRMDNAKFLELPTKVHKWAVLYFHEPRGLVYNEVADFARKFRS**Q**ALTTAVNLEE**Q**AEI**QQ**WKDDR**Q**LDNCFVDLKRDKFDLVIVIIPNRGTTYDTIK**Q**KAELTHGILT**Q**CIK**Q**FTV**Q**RKLNA**Q**LIGNLLLKVNSKLNGINHKLKDDPLTRLVNTMYLGADVTHPSPD**Q**RDIPSVVGVAASHDLYGAAYNM**Q**YRL**Q**RSTAEEIEDMEGIVGEHLRIYH**Q**YHKKYPDHIIYYRDGVSDG**Q**FPKIKSLELRGIYSACAKLKIKPHLCCAIVVKRHHTRFFP**Q**GEPS**Q**YNKFNNVNPGTVVDRTIVHPNEM**Q**FFMVSH**Q**SI**Q**GTAKPTRYNIIENTGNLDIDLL**QQ**LTYNLCHMFPRCTRSVSYPTPAYLAHLVAARGRVYLAGSRLVNSP**Q**SPYSSDVSTK

# Dwil\Ago2b

MVN**Q**VG**QQQQ**TGHGSWAKKEG**Q**T**Q**N**Q**SSWG**Q**KKN**Q**PGPKVN**Q**IC**QQQQ**NGHGSWAE**Q**KV**Q**G**Q**NHSSGGSTLH**Q**PVPPSEGSM**QQQQQ**G**QQ**KAMVN**Q**VG**QQQQ**TGHGSWAKKEG**Q**T**Q**N**Q**SSWG**Q**KKN**Q**PGPKLN**Q**IC**QQQQ**NGHGSWAEKKV**Q**G**Q**NHSSGGSTLH**Q**PVPPSEGSM**QQQQQ**G**QQ**KAMVTDSH

GRTLNYNVEIKETTGFHVDLNSLRSYMNDKIYDKPM**Q**AL**Q**CLEVVLA**Q**PCHKKAIRAGRSFFKAAEPGRRLELGDGYECLIGLY**Q**TFVLGDRPFVNVDISHKSFPIALPVLEYIERYVLKESIKNTTNLDRLYPTIEDFLKGIFVIYEPPSCFNSAPRVFKVNGLTSSSARY**QQ**F**Q**LDNKTLTVE**Q**YF**Q**SRNYSLRYPNLRCLYVGPLDRNIFLPIELCRIEDC**Q**SLNRKDGAN**Q**VAAMIKFSATSTNERKAKIMNLLKFM**Q**HNLDPSISRFGIYIHNDFIVVHTRTLSPPLVEYRNK**Q**MSMVRNGSWRMD**Q**RA**Q**FLEPK**Q**KVHKWAILHSDGRRRLPYN**Q**IADFGNMFRR**Q**GLSVNVCLEEMPDIRCFKDDRELDVYFDDLRRSKCDLVIVIIP**Q**IGVSYDIIK**Q**KAELKHGILT**Q**CLK**Q**LTVERKLNP**Q**LIGNVLLKINSKLNGINHKLKDEPNRLLSNVMFLGADVTHPSPD**Q**REIPSVVGVAASHDPYGAAYNM**Q**YRL**Q**RSALEEIEDMETIVTEHLRIYHSYRKRYPDHIVYYRDGVSDG**Q**FPKIKALELRGINAACAKLGIKPKLCCVIVVKRHHTRFFPTGVPS**Q**ANKFNNVDPGTVVDRVIVHPNEV**Q**FFMVSH**Q**SI**Q**GTAKPTRYNVIENTGNLDIDLL**QQ**LTFNLCHMFPRCTRSVSYPAPAYLAHLVAARGRVYLTGTKLFKSPKEEN**Q**KRLISTSLTTANPMFFV

# Dmoj\Ago2

MGKKNKYKKEEPAPA**QQQQ**AAADSGPGESTPRL**Q**AGDTRGPGP**Q**RSN**Q**PA**Q**GSGH**Q**RPASA**Q**PS**Q**YRP**QQ**AG**QQ**E**Q**GWSRAGPSGA**QQQ**GGN**Q**SGPPRRP**Q**GG**QQ**E**Q**GWSRTGPSGA**QQQ**GGN**Q**SGPPKRP**Q**GG**QQ**E**Q**GWSRAGPSGA**QQQ**GGD**Q**SGPPKRP**Q**GG**QQ**E**Q**GWSRAGPSGA**QQQ**GGN**Q**SGPPKRP**Q**GG**QQ**GGP**Q**SGY**QQ**RGPPGG**QQQ**RGPPGR**Q**GGYD**Q**RS**Q**AGAMGPPLP**Q**TGNIKR

GTLGRAGFVDINYLDVDISKMPDIAYHYDVSIVPERPKKFYRNAFEEFRTKHLNNAIAAFDGRKSCYSVDKL**Q**NTTGEVVDRHGRTVRYTLTIKETDNSEVELSSLRSYMNDKIYDKPMRAL**Q**CLEVVLAAPCHSTAIRAGRSFFKNSNEGERYELGDGYEALVGLY**Q**SFVLGDRPFVNVDVSHKSFPIAMPMIEYLERFALRSKINP**Q**SMLGNTY**Q**LMNFIKGINIVYEAPASFATAPRVYKVNGLSP**Q**PANE**Q**KFKLDDKTMTVSEYFRSRNYNLKYPKLHCLHVGPPAKNIYLPIELCRIEEG**Q**ALNRKDGAN**Q**VSEMIRFAATSTDERKKKIMDLLKYFHHNTDLTISRFGIRISSDFITVNSRLLTAP**Q**LEYRNSKFALPSNGAWRMDRC**Q**FLKPTPKAHKWAILHCEGARLLYNKVCEFEKMVVN**Q**CNDVNVSLEPRAEIRTFKDERNLDDHFKELKLNRFDLVFVIIPSRGATYEVIK**Q**KAEL**Q**HGILT**Q**CIKHNNVDRRLNA**Q**LVGNILLKVNSKLNGINHKLKDDPRT**Q**LTNVMYLGADVTHPSPD**Q**RDIPSVVGVAASHDAYGGSFNM**Q**YRL**Q**CGAGGRAAEEILDMESIVTEHLRVYKEH**Q**KRYPDHIIYYRDGVSDG**Q**FMKIKNIELKGIYVACTKLGIKPKMCCIIVVKRHHTRFFPKG**Q**PHPRNKFNNVEPGTVVDRTIVHPNEM**Q**FFMVSH**Q**SI**Q**GTAKPTRYNVIENTGNLDIDLL**QQ**LTYNLCHMFPRCNRSVSYPAPAYLAHLAAARGRVYIYGSRLCRSPEEEYKRRLIVPDFLKTNPMYFV

# Dgri\Ago2

mgkknkykkdekeaaapkpaplp**qqq**a**q**p**qq**p**qqq**k**q**r**q**tvtaataptpstsaaaa**q**a**q**g**q**sggtvtpgh**qq**sg**q**p**qq**gk**q**gg**q**ygg**qq**gagy**qqqqqq**r**q**sgg**qq**ggy**qq**rggagdgrgppgy**qq**gg**q**p**qq**gr**q**gg**q**sgg**qq**ggy**qq**rgggadgrgppgy**qq**gg**q**p**qq**gr**q**gg**q**ygg**qq**gagy**qqqqqq**r**q**sgg**qq**ggy**qq**rgggadgrgppgy**qq**gg**q**p**qq**gr**q**gg**q**ygg**qq**gagy**qqqqqq**r**q**sgg**qq**ggy**qq**rggagdgrgppgy**qq**gg**q**p**qq**gr**q**gg**q**ygg**qq**ggy**qq**rgatp**qq**ggamvsplp**q**tgtikr

gtigrpgtvgvnyltvdlskmpkfayhydvditperpkkfyrdafevfrtkylk**q**siaafdgrkscyalddlpsvsgevevvdrngrtlkytvtlkktdnsevdlsslhsymndkiydkpmral**q**clevvlascch**q**rsvragrsffktsgnacyklddgyealvgly**q**slvlgdrpfinvdvshksfpmamtlieyleeyglmeplrentti**q**fns**q**rkii**q**flkginvvyeppksfgnaprtfrvngis**q**nparsekfkcgevvmtveeyfksrsyklkypml**q**clsvghptnvlllpmelcrieeg**q**alnrkdgtn**q**vskmirfaatatherkakildllkyf**q**hnldptisrfglrigddfitvntrlliap**q**veyrnskfatpmngswrmdrn**q**fltrgpkvhkwailhceaarm**q**ynkimdl**q**nmvlk**q**akfvditlepaaeirsfkddrnldipfndlkknkydlvfviipsrggasyemik**q**raelhcgilt**q**cik**q**nnverrldd**q**lvgnlllkvnsklnginhkikddvriklpnvmylgadvthpspd**q**rdipsvvgvaashdlyggsynm**q**frm**q**snsggrpreeiddm**q**siasenlrvyf**q**k**q**grypdhiiyyrdgvsdg**q**flkiknielrgiyaacekvrikpkmccvivvkrhhtrffplgtphprnrynnvepgtvvdrvivhpnev**q**ffmvsh**q**si**q**gtakptrynvventgnldidll**qq**ltynlchmfprcnrsvsypapaylahlaaargrvyitgsnrpn**q**spvinaefmktnpmyfv

# Dvir\Ago2

MGKKNKYKKDDKEAAAPAP**QQ**R**QQQQ**TPAPSTSAAARENTSRL**Q**AGDDGVPAT**Q**RP**QQ**HPES**Q**NP**Q**GEWRRP**Q**GG**QQ**RGP**QQQQQQ**YRP**QQQ**GG**QQ**R**Q**LGWK**Q**ESSG**QQ**GGY**QQ**RGPSSGP**Q**GGA**Q**GGG**QQ**GGY**QQ**RGPSSGP**Q**GGA**Q**GGG**QQ**GGG**QQ**GGY**QQ**RGPSSGP**Q**GGA**Q**GGG**QQ**GGY**QQ**RGLSSGP**Q**GGT**Q**DGG**QQ**GGY**QQ**RGPSSGP**Q**GGA**Q**GGG**QQ**GGY**QQ**RGPSSGP**Q**GGA**Q**GGG**QQ**GGY**QQ**RGPSSGP**Q**GGA**Q**GGG**QQ**GGG**QQ**GGY**QQ**RGPSSGP**Q**GGA**Q**GGG**QQ**GGY**QQ**RGPPG**QQQ**GGY**QQ**RGP**Q**GP**QQ**GGY**QQ**RGPPPA**QQ**G

GY**QQ**RGPP**Q**G**QQ**GNIKRGTIGNAGRVGVNYLDVDISKMPDIAYHYDVTIVPERPKKFYRKAFEVFRAKHLDNGIAAFDGRKSCYSVDKLPNVTG**Q**VEVIDPHGRTVRYTLTIKDTDNSLVELSSLRSYMNDKIYDKPMRAL**Q**CLEVVFAVPCHTRSIRAGRSYFKTSDNGERRDLGDGYEALVGLY**Q**SFVLGDRPFVNVDVSHKSFPIAMSMIEYLERYGL**Q**SAITPATTLNNTLRLIPFIKGINVVYEPPKCFGSAPRVYKVNGLSEKPANE**Q**KFELEGEVKTVD**Q**YFRSRNYTLRFPKL**Q**CLHVGPPSKYIYLPMELCRIEEG**Q**ALNRKDGTN**Q**VSEMIKFAATSTNERKAKILDLLRYFKHNSDPVISRFGIHVAGDFITVSTR**Q**LPAP**Q**LEYHKKNFARPFNGSWRMDNL**Q**FLVVKPKTHRWAILHFEGARMHYNKVAEL**Q**DLVI**QQ**ARYVNVCLEPKADIRTFGDARNLDVHFKDLKD**QQ**YDLVFVILASRGPSYDIIK**Q**KAEL**Q**YGILT**Q**CIK**Q**NNVERRLNG**Q**LVGNILLKVNSKLNGVNHKLKDDPRTMLANVMYLGADVTHPSPD**Q**RDIPSVVGVAASHDMHGGSYNM**Q**YRL**Q**CGGGGGAREEIEDMESIVTEHLRVY**QQ**Y**Q**KRYPDHIIYYRDGVSDG**Q**FLKI**Q**NIELKGIYVACTKLGIKPKMCCIIVVKRHHTRFFPEGMPSNPRNNKFNNVEPGTVVDRTIVHPNEM**Q**FFMVSH**Q**SI**Q**GTAKPTRYNVIENTGKLDIDLL**QQ**LTYNLCHMFPRCNRSVSYPAPAYLAHLAAARGRVYITG

# Cqu\Ago2-1

MESAKPKGNYRGKKKP**Q**SGD**Q**DVPEGTSG**Q**TAGPP**Q**G**QQQ**K**QQ**KSG**Q**GGVGKYK**Q**K**Q**LLK**QQQQQ**DL**QQQ**TGEPIEKSLAKVKLDFV

RPKNYGVAGTPVKLEVNYLALNLDKLPAKAYHYDVDI**Q**PAASRKW**Q**RACFSGFRAEALPNRLIAYDGHKNAYTM**Q**PMD**Q**MDKVGVAVSLDNRERRFTVSVKLANVVDLRSLKGGNEHN**Q**APAK**Q**CLEVVFGSKLFLNIDVAHKAFPSGVPVLDVVGDLARRRWNDSPNVPERIDDTLAFKLHNFLKGLEVSYTGPSSVKKVFKYNSLRGPASS**Q**LFKREDGTKMTVAAYFT**QQ**GYRLRHPELPVMHVGSIVRNIMLPMELC**Q**ILPG**Q**ALNKKHPDECTA**Q**IIKRAATDAPTRKRKIMELRD**Q**ISYSNCPIIKEFGIGVGKDFEVIDGRIIAPPLIEYKNRRTVLPEHG**Q**WSADNEGFITSN**Q**RELRWIILNLDSYDTR**Q**RDVDSFGNNVFNESRKKGM**Q**LEPFSM**Q**NNYYEPRNTRMNMK**Q**LETELENSLGYFKK**QQ**LDFVIVVIPGIGDHYSRLK**Q**KAELVVGVLTSCVKGNTVKNTRSPLTVVNNILLKINGKTNGTNHVV**Q**SPDPKIPLIKKRIMFVGADVTHPSPE**Q**STIPSVVGVVASFDRNGFRYKPHF**Q**L**Q**DPKKEMIHGLEAIM**Q**AMLNNYKNKNN**QQ**LPEMILYYRDGVSDG**Q**FS**Q**VLDIELNAINRAVAAMNPPSKINVTFVVV**Q**KRHHTRFFPGPKCPKEGRN**Q**NVPPGTIVDRYITTPKHF**Q**FFLTSHRAVEGVAKPSKYTVLHDDE**Q**WDPDRL**Q**AITYALCHMYARCNRSVSYPAPTYYAHWVAARGKVYI**Q**GRTLNMAELDRENSLLRIRPEIIGERSMFFI

# Cqu\Ago2-2

MGNSMEVVATETAPEVEEEAKAKVVAVARRVNSSSNSPNMVKVNSRDKEKGSTSK**Q**K**QQQQQQ**P**Q**A**Q**PPSEPAGGS**QQ**YGDFL**Q**KM**QQQQQQ**VA**Q**PAPAPA**Q**A**Q**AEPPK**Q**A**Q**G**QQQ**KGGGGGGKYK**Q**K**QQQ**K**QQ**E**QQ**ELEK**Q**LAA**Q**RP**QQ**PPAAASAA**Q**SRSTASPEGSLSPTHGAI**Q**RVEEDLSGMKIDKGSGKSALR**Q**VLIRPGAH

GRRGKVTKLEVNYIPLMLEKMVSTAYHYDVDI**Q**PMASRKW**Q**RAAFKRFEVEALAG**Q**PIAFDGNKNAYTAKKLKLDHYKKEVVAREDNRDRKFTITMKEAAVFRKSIYVVPTKPEDIGANHELWYGLF**Q**SALLGSKPFLNIDVSHKAFPRGGPVLDVVASLNRNSLPTTLAGWLA**QQ**VHDYLKGMEVVYTGPNGVGKTFKYNSLKGPAST**Q**KFKLEDGSESTVAAYF**Q**K**Q**GVRLRFPDLPVMHVGSTIRNIMVPMELCAIPPG**Q**ALIKKHPD**Q**CT**Q**MIIRRSATDTVTRKGKIMDIFN**Q**IDYNNCKTIKDFGFGVGNSFEVVDGRIIGPPSVVYRNNVTITPSRG**Q**WRADNASFI**Q**INP**Q**PLRWRILNLDDRTRPAGI**QQ**FG**Q**NIF**Q**VSRKHGI**Q**LEPFSM**QQ**TYYEPRDLRYAIREVDSIFEDLKK**Q**RMDFVIVVISGMGD**Q**YSKVK**Q**RAELVTGLLT**Q**CIKGDTVFKKAGDMSTINNIWLKINAKTNGTNHVLKPESKPPLIRKRVMYVGADVTHPSPE**Q**TNIPSVVGVAASYDLEGFRYNCCYRL**Q**NPKDEMIRDLENIIKK**Q**LL**Q**FKTCNGALPDLIMYYRDGVSEG**Q**FSEILTIELNAI**Q**SAVASTSPGVKVAVTFIVV**Q**KRHHARFFPTRGTIEVEGRN**Q**NLPPGTVVDKHITAPN**Q**Y**Q**FFLISH**Q**AV**Q**GVAKPTKYCVLYDDVNSDPDEL**Q**SVTYALCHMFARCNRAVSYPAPTYYAHLAAFRGRVYIKDRRLNMNDLAGEYRKM**Q**IKPEIIDGHPMFFV

# Aae\Ago2

milnarylyipcr**q**k**qqqqqqq**p**qqqqq**h**qq**k**qq**s**qqqqqqqqqqq**rske**q**gs**qqqq**rp**qqq**a**qqqq**ps**qqqqq**s**q**k**qq**hp**qqqqqq**rp**q**k**qqqq**f**qq**d**q**rp**qqqqqq**l**q**k**qqqqq**g**q**swrp**q**shdpspasgshshssspshaaalerveedfskikidk**q**kihssallpvlm

rpnahgtrgraikvevnyi**q**lllerliptayhydvdi**q**paasrkw**q**rlafseftk**q**mfpnhgfafdghknayaarrl**q**advye**q**evkvrdegrerrfkvamkeaavldmtclktymnngstldkpmsai**q**cldivlrtayennprfikfkksiyvkpdrpddigsnhelwyglf**q**sallgarpflnidvshkafptggpvlrilvdmnrg**q**vpdrvtdwmsrdlhdflkgmelsytgpdgvsklfkynsikspan**qq**kfklengtemtid**q**yfrsknk**q**lrypslpvlhvgslvrnvmlpielcsippg**q**alnkkhpd**q**ct**q**fiirksatdtatrkrkimdlfn**q**igynnaptikefgvsvgnnfetvdgrildppelsyrndrrvkpmrgvwradnmnfiipsteitrrelswtilnldgrtrpdaidefgrniy**q**mslk**q**gv**q**l**qq**fsmknnfyeprdmrfavkdldnifdelkkrkidlvfvvipspgrdgdvyakvk**q**kaelcvgllt**q**ciksftldkkrgdmstisniwlkinaktngsnhvlaknfkppiarktvmyvgadvthpspe**q**tnipsvvgvaasydlegfrynccyrl**q**gpkdemirdl**q**nivik**q**lr**q**fk**q**tn**q**slpelimyyrdgvseg**q**f**q**evltielram**q**aaaasv**qq**gykpnitfivv**q**krhharffptancptegrnnnv**q**pgtivdryitapn**q**y**q**fflvshaav**q**gvakptkycvlyddencnpd**q**l**q**altyylchmftrcnravsypaptyyahlaayrgrvyikdrplnmnnltkeyerm**q**irtei**q**dghpmffv

# Amel\Ago2

markgkkk**q**nv**q**nhphssis**qqq**snpd**qqq**npsgs**qq**vhs**qqqq**sdis**q**khacpk**qq**sdsp**qqq**g**q**tyd**qqqq**hsss**qq**shs**q**k**qq**snsp**qqq**awkln**qqqqq**ssp**qq**phs**q**r**qq**ldsp**qqq**awrln**qqqqq**ssp**qq**phs**q**r**qq**snsp**qqq**vrrpn**qqqq**yssp**qq**shs**q**r**qq**snsp**qqq**awkln**qqqqq**ssp**qq**phs**q**r**qq**snsp**qqq**awrln**qqqqq**ssp**qq**phs**q**r**qq**lnsp**qqq**awrln**qqqqqq**ssp**qq**phs**q**r**qq**snsp**qqq**vrrpn**qqqq**yssp**qq**shs**q**r**qq**snsp**qqq**awkpn**qqqqq**ssp**qq**phc**q**r**qq**snsp**qqq**vwrpn**qqqqq**hnsp**qq**vsish**qq**imsdsyee**qqq**snkpkf

llrtvfeefrkk**q**cpkrypafdgrknaysakllpfgdkskeeeinvfdvntrkernfkiylnkvacldlswltnlkcdmmdsern**q**kci**q**aldiilrhgpay**q**ytvvgrslf**q**ppepgrivslsngldlwvgvf**q**svvigskpylnidvahkgfpks**q**svielmkelfdsarenkfrled**q**tlcsvekyfl**q**ikkytikypnlpclwvgs**q**knsiylpaelctviag**q**vinkemnki**q**tskmvretatnt**q**krkekimngykekeitvskgtwkadkffspcvlpknlwtilnldkfvnahdlynlhnkllhsvvilpnldnaysivk**q**isel**q**ihegivt**q**cikn**q**tlkklndstignillkinsklnginhiitptnrpncly**q**pcmiigadvthpspdatnipsiaavaashdpnafkynveirl**q**spreeii**q**dleeimii**q**lkyfyvttg**q**kp**q**klifyrdgvsegelvkimhkelsaikraiarleksnelripitflvv**q**krhhvrffptdaknsddknfnv**q**agtivdteithpthidfylvshasi**q**gtarptkyrcicnen**q**mpeneieeltyylchmfarctrsvsypaptyyahlaafraralihk

# Nvit\Ago2

mgkggkkkk**q**stedspsgsts**q**d**q**agps**q**s**qq**pp**qq**s**q**p**q**sgrgrgrdlrnpvers**q**pnvta**q**sap**q**p**qq**p**qq**s**qqq**sa**q**dtagrgrgwg**q**grg**q**grg**q**grgrggfsnpvvgs**q**pnvpp**qq**tpwgr**q**p**qq**pa**qqqq**hsa**qqq**kpp**qqq**e**qqq**rpse**q**hsvgsketkaadvk**q**tteels**q**lslkepkhslpkse**q**vvpiltvpaniani**q**a**qq**ipkrknpmka

gtkgrpitvktnmmainvr**q**mnsnvvhydvdivpntpkylmrpvfleakkklfpnrnpafdgkknafsagdlpikdpstaevvvynedgrekkytvtmkianridlswlktvkpgl**q**etern**q**isl**q**aldvimrnapaltstpvgrsfftppkg**q**vmslgggmdlwvglf**q**savlgwkpylnidvahkgfpkp**q**svldlmkticgcdg**q**d**q**ggr**qq**ygr**qq**gygr**q**ggyg**qqq**gpatlsadli**q**rnredikkflkglkvtieipg**q**ptsrrt**q**rvndlvkpprdnvfehnghkitve**q**yykhekkytikypdfpclwvggkdknvhvppeictivgg**q**at**q**kklden**q**tssmikfaatgtedrkrkimdafnsmrhn**q**dpcmkefgisvsgefetvparvldpp**q**lry**q**r**q**narvakgvwras**q**fikpspligedntwtvlnldyrtrddglyklvdtlkrtg**q**tlgmpvgnplspfrsm**q**lrg**q**dmrelmayfnemktk**q**iklvvvvvpemkgpyskvk**q**mselrvgvlt**q**clksktlfklndatagnillkvnaklngtnhifeetvsrppclkrpcmivgadvthpspdatdipsiaavaashdpnafkynveirl**q**ppk**q**eiigelaeimki**q**lkyfytstgykpekiifyrdgvseg**q**fg**q**imhaellairkac**q**sl**q**adykpkitllvv**q**krhhirlfptdprnsddrnfnv**q**agtivdteithpshidfylvshasi**q**gtarptkyrclwddsdmsedeienltyflchmfsrctrsvsyptptyyahlaafraralt**q**dvdidmnnlp**q**e**q**lrkltikdevlkgspmffv

# Bmor\Ago2

margknkggkkeapdstktpsses**q**pses**q**psts**q**iptteptttieddlgglglgesrkrrprkkptek**q**esla**q**aelsnpklt**q**tdnpkaevpkteapktealkpeapipeackseapkseeskietrgskpeaaadkp**q**ddddglglglggggrkktrsrkpkftavetdikyskapssepaipgps**q**skpitstas**q**pi**q**yv**q**nkpevkaapaapvlykipdkilspps

rtvpiltnylamkitkplkiyrydvtfkpdkpkkfia**q**vfklvkskefpkeilafd**q**tkncysltplpkitterygvkvvikdmngkdmpfevsfkasgivdynnvlkhmatggsslnaptdti**q**cidivlk**q**gtlesyvkagr**q**yfmrpaspidlgdglemwtglf**q**saiftskafinvdvahkgfpkn**q**pmidaftrdfrldpnrpvdr**q**pgraaeafnefirglkvvskilgtgpssg**q**lrehicngvvdppsr**q**tftlendkgppvrmtvyeyfmkekkyrikypdlnclwvgpkdkniylpmelvevayg**q**arnk**q**lndr**q**lstmvreaatppdvrkrkieevi**q**kmnyskn**q**ffktygleianefy**q**veakileaptlevgpr**q**ftvpkkgvw**q**ancllkpealnswgfiaieldprgcnyedivsklmntgr**q**mgmnvt**q**pkmacfnirindlhksmlhalek**q**vnflvvvvsgrgrdyyhklk**q**iaelkvgilthvfkedtatrrmnp**q**tarnillkvnsklmgin**q**alenrsip**q**clkggavmivgadvthpspd**q**snipsiaavtasmdtkcyiynielsi**q**tpkkemiv**q**fedimvdhfhafkks**q**gilpkkvfvfrdgvseg**q**faevmkseltglhray**q**rvaglnakpevlfilv**q**krhhtrfflpgnnarfnvdpgtvvdrdivhpreldfylvsh**q**aikgtarptryhavcndgripenevehlayylchlyarcmravsypaptyyahlaclrarsltygeifnnndleknpkrlrvldsmlk**q**srmffv

# Tcas\Ago2a

maplpdgpdpstk**q**k**q**pptfap**q**kessfst**q**klksslsdcls**q**tviikp

gvkgrpikiesnhlslnvgtlteayhydvsitpdtpksflrdvmnlfarkhypknhpafdgrknlyspkklplpndtmsdtielegenkkrgfkvvvklartvdlsplrdil**q**tr**q**sp**q**dal**q**cldivlrnapsnscissgrcfftppreg**q**ilrlgdgmemyygfy**q**sairgwk**q**pllnvdvvhkafpealnvldlvcelgsdyrntmtr**q**dln**q**pltdfv**q**kalekflk**q**lkvtyeipg**q**sgsrrifrvnglrapps**q**arftlgdgkvttvekyy**q**evkrcrl**q**yphlptlwvgsr**q**revliplefctvvsg**q**vvnrkmnen**q**tsvmikkaatstdvrkdkim**q**vlrkanynsdpcvrefgfsvnnsfekldgrvl**q**pptllyarkaevtpskgvwradmnrffvgaivhkwtivsctrhperge**q**ladmifrmassngm**q**itskatgpf**q**hlggr**q**nlrdiidyfkrk**q**dhdliivvvpnsgp**q**yslvk**q**aaelnvgclt**q**cikertiaklnp**q**iianillkinsklngtnhilssrlpimsrpciimgadvthpgpdakdvpsvaavtashdpnaf**q**ynicwrl**q**ppkveiiedlcaitve**q**lmffyrktrhkpetivffrdgvseg**q**faevrraeisaih**q**ackkl**q**regyepritflvv**q**krhhtrlfptnprdsedrnnnvpagtcvdthitnpmm**q**dfylvshasi**q**gvakptkyctlwddnnmsnddieeltyylchmftrcnrsvsypaptyyahlaaarakvyvenvkldlt**q**lkth**qq**kc**q**i**q**esivkekpmffv

Tcas\Ago2b

mgap**qqqq**p**q**rgplp**qqqqqqqq**rrp**qqqq**ppvkspvphpepssppr**q**epapplsgggdclsgalvvtp

gtkgrri**q**iesnhlslnlgklteayhydvaitpdtpkcllrdvmnlfgrkhyp**q**nhpafdgrknlyspkklpfpndtksdtievegenrkkefkvevklartvdltplhdimrtt**q**sp**q**dal**q**cldivlrnapsnaciiagrcfftpprdg**q**iiplgdgmelyygfy**q**sairgwkallnvdvahkafpkasnvldivceigsdfrttmtranls**q**plrefv**q**rdfekfik**q**lkvkyeipn**q**ssskrihrvnglgepps**q**akfklddgrmttveryy**q**evkrckl**q**yphlptlwvgsrereskillplefctvvgg**q**ainrkmnen**q**tsamirkaatstdvrkdkim**q**tlrtanynndpcirefgfsvsnnfekldarvlnppsllyadna**q**ikpskgvwradrnrflvgatinkwtiasgtrypsrdadkladmifrmassngm**q**itskatpsthiggr**q**glrdfidyfkgk**q**dydliivvvpnsgp**q**ysfvk**q**aaelnvgclt**q**cikertigrlnp**q**tvgnillkinskmngtnhrlspnsrplimkrpcmimgadvthpspdardipsvaavtashdpnaf**q**ynicwrl**q**ppkveiiedlcnitve**q**lkffy**q**ktgfkpesivffrdgvseg**q**fk**q**v**q**raeiaai**q**kackml**q**kddyepkitflvv**q**krhhtrlfptnprdsedknnnvpagtcvdthitnprm**q**dfylvshasi**q**gvakptkyctlwddnnmnnddieeltyhlchmftrcnrsvsypaptyyahlaaarakvyiendkldms**q**lkrh**q**ekc**q**i**q**ekivkgkpmffv

# Apis\Ago2

mnpn**q**pntgtpgvpgkkk**q**kkgv**qq**dssntnvg**q**s**q**epsnsgskdfp**q**is**q**skpnkk**qq**ap**q**k**q**nkpkdan**q**kpn**q**k**q**n**q**saepk**q**tkk**q**n**q**pggtkpn**q**k**q**segnnpsaavpksspt**q**gg**qq**ie**qq**ivilksnlhipkrknpktg

gslgrateievnhlplnld**q**lfkkvvyhvdv**q**ftpelpkrllrnaleefnnrhypkvnfafdgrrnmytikeikgksdtvsvvndennrtidfgistsivntihmnkiedylksgssntppgeaf**q**aldivlknrpfalrftnvgrsffpvpritpvdlgegmelwkgff**q**spvmgwkpylnidvahkgfpky**q**plinfitnemncdlnsemd**q**rsyntlasyvkglkidftvpn**q**pntkrsykvvglldtasrfrfemedpvrgk**q**tlnvv**q**yfritrnyvikhpnlpclhvgnvnkktaipielchv**q**kg**q**lrlkklsei**q**taamvknaarppgerr**q**tiencirdiaynkdpvlkdfgievkehfasiparvld**q**pslaya**q**nketkpragvwrpdrfskavhinkwvvlnld**q**rtniasiknfekslmmsardlnvvmspmdpviniflprssladiktsignvftk**q**kacntelivvvipdypagiyasvk**q**kselevgilt**q**ciksktmfrmntstssnillkinsklnginhtlairssppsmegaiifgadvthpspe**q**ttipsvaavtashdtygs**q**ynmewrl**q**spkveii**q**dledivhi**q**llkykertktvpkkifyfrdgvseg**q**fl**q**lleyeliairraclrlniaykpsvtflvv**q**krhhtrmfpkfsydmdgkfsnvpsgtiidt**q**ithpteldfylcshasi**q**gtsrptkyhliwddnnfted**q**le**q**ltfylcfmfvrctrsvsypaptyyahlaafrarayienktinlnnlede**q**trn**q**lnhsftvntpmffv

# Phum\Ago2

Mtekkwyhkknkkdktehhadkhkpgfsgds**q**pvagetssfekvssekmpksrseidfdkk**q**emp**q**sfkh**q**k**qq**lsstekegvesh**q**k**qq**psdyskset**q**i**qq**khknpk**q**kmkv**q**r**q**pdlpisek**q**ppsa**q**gak**Qq**p**qqqqqq**s**q**ssm**q**ppsawgvk**Qq**p**qqqqqqq**p**q**ssm**q**ppsawgvk**Qq**p**qqqqqqqqq**ssv**q**spsawgvkE**q**pkyknskgserffedskykkgwdkkntak**qq**lssaeeggdeph**q**k**qq**psdsskset**q**f**qq**k**q**knpk**q**kmkv**q**r**q**pdlpisek**q**ppsa**q**dak**Qq**p**qqqqqqq**p**q**ssm**q**ppsawgvk**Qq**p**qqqqqqq**p**q**ssv**q**ppsawgvke**q**pehknskksekffkdsknkkvgdtknipkegkgdsrdpsakstelit**q**kldkmnlekg**q**egrrspnspdk**q**lipfa**q**kivd

prkdgrrilvdtnyfplrisnpnvivyhydvvfnpdtpkfmlrlawnkyasdnfknritvfdgkknayskgplisngnchsagvsivnpnsgkirefsvsi**q**evr**q**dyk**q**slehnvklpeaeisvleiilkngpsntlvpcgrsffpks**q**mgirarsisscldyraghy**q**aatlgdriylnfdla**q**kafykslpvidvaysftssynnrinldslnyflkgakveyklpnssdpgkiykvnglvgnaeeetfekdtitmtvatyfakdkkyplkhshlpllwvgst**q**rkiylpmefcrived**q**vfmgemspdetremvklatsdaktrkkdilyvfnkanfsnniymkefglsvdkdmeri**q**arileppslrtkgsvfvkdg**q**wkirdfftptvldnwcilyfdncvrkenlndf**q**nlikrkgtefnm**q**vkdairnpvggnshprelartl**q**elk**q**mklvfviipetkglyskiknicet**q**egilt**q**cvrartlc**q**dfrkfsttvenillkvnaklggrnvsldknsfptcleglvmvigadvthsgpgsripsvaavavscdp**q**af**q**ygistrv**q**igeii**q**dmeniigehlrkfy**q**ir**q**nypekiiffrdgvsegelpimmkteleaikracsrvpscrpkitmivv**q**krhhtrfypvndadaigrnfnvpvgtcvdtdivhprdldfylvshasilgtarptryrllyddnrltddeiekmsfylchmfsrcnrsvsypsptynahlaafrvraylearnlsknddlksi**q**aklkdvis**q**knpmffv

# Dpul\Ago2

MGRKRK**Q**TSGG**Q**EGAET**Q**AATEME**QQQ**PPA**Q**AMA**Q**LNLGS**QQ**LPAGL**Q**AMP**Q**HPAGP**Q**VMP**Q**RPAGP**Q**AM**QQ**RPAGPLAM**QQ**RPAGPLAM**QQ**RPAGPLAM**QQ**RPAGPLAM**QQ**RPAGPLAM**QQ**RPAGP**Q**AM**QQ**RPAGP**Q**AMP**Q**HPAGP**Q**AM**QQ**RPAGP**Q**AM**QQ**RPAGP**Q**AM**QQ**RPAGP**Q**AM**QQ**RPAGP**Q**AM**QQ**RPAGPPNV**QQQ**R**Q**PGPG**QQ**AM**QQQ**RPNAPTTS**Q**FP**Q**GMA**Q**RPGTP**QQ**PRPAAP**Q**T**QQ**PRPAAP**Q**AA**Q**SRSITP**Q**AHRLVA

**Q**GAALRPPPRSAAGGEGTLGRPIKLSANHFAVIMKKPILYHYDVEVKPLPPKALFKKVIV**Q**FLESEARFKDIFPVFDLKKNIYTARRIPGLDSKVDIKFEF**Q**ELDRETPRLNEFIISL**Q**PTGEVEIDVGALASYC**Q**GGSGSSVDIPLRPI**Q**ALDIALKYGAA**Q**RPTKVMLGSCLLSKPVGRSEDLGGGVEVWFGHF**Q**SLRLGWKPFLNVDAT**Q**RAFLRSGLVHDIMADMFRARPGDRLDDRDYGDFHKKIATLKVSYNRGKYIATVGCNGIKGAANTEKFECDGKTITV**Q**EYFEKKLNTKLKYPHLPCVWVGSREKKNLVPMELCSIAEG**Q**EYRRKLTDF**Q**TSAMIKVAATPADVRKRKILDSVNGM**Q**FA**Q**D**Q**YA**Q**HFGRVLPTPKLVYGDKECSSIVPRDGVWNMRNMKFIEAKAMNSFGLINITRCGDREIDFFISALTKAGREMGMSMG**Q**LLFNRPCGIRDLESTMKMAK**Q**KFP**Q**L**Q**IIFVIINRKGDPAYEIVKRVGDLDLKITT**Q**CI**QQ**KNVTGRNGPDPSTMANICLKLNAKLGGINNLISRDFRPKMLLNE**Q**VIIMGADVTHPGAD**QQ**DSGKPSIAAVVGSVDPRAS**Q**YCCEIRI**Q**KSK**Q**EYIEDMENMVYNLLRKFNRAAGATSTGKP**Q**RIIFYRDGVSEG**Q**FAKVLEWELSAIRKACMKLEVGYNPPVTFIVV**Q**KRHHTRLFPED**Q**RDECGRGKNVPPGTIVDNTIVHPVE**Q**DFFLVSH**Q**GI**Q**GTSRPTHYHVLWDDSKF**Q**ANDI**Q**MLTYYMCYLFTRCTRSVSYPAPCYYSHLVAFRGR**Q**YYDNLTGNR**Q**AVSSTAL**Q**THIDSTRDLSFIYRVSLIEH

# Hsap\Ago2

mysgagpalappappppi**q**gyafk

ppprpdfgtsgrtikl**q**anffemdipkidiyhyeldikpekcprrvnreivehmv**q**hfkt**q**ifgdrkpvfdgrknlytamplpigrdkvelevtlpgegkdrifkvsikwvscvsl**q**alhdalsgrlpsvpfeti**q**aldvvmrhlpsmrytpvgrsfftasegcsnplgggrevwfgfh**q**svrpslwkmmlnidvsatafyka**q**pviefvcevldfksiee**qq**kpltds**q**rvkftkeikglkveithcg**q**mkrkyrvcnvtrrpash**q**tfpl**qq**esg**q**tvectva**q**yfkdrhklvlryphlpcl**q**vg**q**e**q**khtylplevcnivag**q**rcikkltdn**q**tstmiratarsapdr**q**eeisklmrsasfntdpyvrefgimvkdemtdvtgrvl**q**ppsilyggrnkaiatpv**q**gvwdmrnk**q**fhtgieikvwaiacfap**q**r**q**ctevhlksfte**q**lrkisrdagmpi**q**g**q**pcfckya**q**gadsvepmfrhlkntyagl**q**lvvvilpgktpvyaevkrvgdtvlgmat**q**cv**q**mknv**q**rttp**q**tlsnlclkinvklggvnnillp**q**grppvf**qq**pviflgadvthppagdgkkpsiaavvgsmdahpnrycatvrv**qq**hr**q**eii**q**dlaamvrelli**q**fykstrfkptriifyrdgvseg**q**f**qq**vlhhellaireaciklekdy**q**pgitfivv**q**krhhtrlfctdknervgksgnipagttvdtkithptefdfylcshagi**q**gtsrpshyhvlwddnrfssdel**q**ilty**q**lchtyvrctrsvsipapayyahlvafraryhlvdkehdsaegshtsg**q**sngrdh**q**alakav**q**vh**q**dtlrtmyfa
