# Supplementary material for: Natural Variation of the Amino-Terminal Glutamine-Rich Domain in Drosophila Argonaute2 Is Not Associated with Developmental Defects
Source: PLoS One. 2010 Dec 17;5(12):e15264. doi: 10.1371/journal.pone.0015264 (PMC3002974; doi:10.1371/journal.pone.0015264)
Supplement: Figure S5 — Comparison of Dpse\Ago2c and Dper\Ago2c. Dpse\Ago2c and Dper\Ago2c were aligned by CLUSTALW. The two proteins are almost completely identical through the entire coding region. The major differences are in the center of the NTD, where alignment is spotty, suggesting rapid changes in primary sequence. Color code for domains as in Fig. S4. (DOC) [file pone.0015264.s005.doc]

**Figure S5:**

Dpse\Ago2c MGKKNKYKEAEKPVPPAAQPQQQQQAAAAPGAQNRPTQTPSTSSAASGSQQQQGGWRTQD 60

Dper\Ago2c MGKKNKYKEAEKPVPPAAQPQQQQQGWQQQGGGQQ------------RGPQQQGGGQQRG 48

*************************. *. :: . ***** : :.

Dpse\Ago2c SHQQRSQAGQGWQQQGGGQQRGPQQQGGGQQRGPPQQQGGYQQRPQGQQAQ 111

Dper\Ago2c PQQQGGGQQRGPPQQQGGY-QGQQAQG--QYRGPPQQQGGYQQRPQGQQAQ 96

.:** . :* ** ** :* * ** * ********************

Dpse\Ago2c GQYRGPPQQQGGYQQRPQGQQAQGQYRGPPQQQGGYQQRPQSQQGRVQGGAALPPLPAGT 171

Dper\Ago2c GQYRGPPQQQGGYQQRPQGQQAQGQYRGPPQQQGGYQQRPQSQQGRVQGGAALPPLPAGT 156

************************************************************

Dpse\Ago2c MKRGTLGKPGQVSVNYLDVNLDKMPAVAYHYDVKITPERPKKFYRQAFDQYRVEHLGGAI 231

Dper\Ago2c MKRGTLGKPGQVSVNYLDVNLDKMPAVAYHYDVKITPERPKKFYRQAFDQYRVEHLGGAI 216

************************************************************

Dpse\Ago2c AAFDGRASAYSAVKLKCSSQGQEVKILDRHGRTLTYTVELKETEDLEVDLNSLRNYMKNK 291

Dper\Ago2c AAFDGRASAYSAVKLKCSSQGQEVKILDRHGRTLTYTVELKETEDTEVDLNSLRNYMKNK 276

********************************************* **************

Dpse\Ago2c IYDKPMRALQCLEVVLAAPCHNTAIRAGRSFFKRSEPGKAFDLNDGYEALVGLYQTFVLG 351

Dper\Ago2c IYDKPMRALQCLEVVLAAPCHNTAIRAGRSFFKRSEPGKAYDLNDGYEALVGLYQTFVLG 336

****************************************:*******************

Dpse\Ago2c DRPFVNVDISHKSFPKAMPIIEYIEQYQRQKIDKSTNLDYRRYDIESFLKGMNIIYDPPA 411

Dper\Ago2c DRPFVNVDISHKSFPKAMTIIEYIEQYQRQKIDKSTNLDYRRYDIESFLKGMNIIYDPPA 396

******************.*****************************************

Dpse\Ago2c CLASAPRVFRVNGLTKFPASSLKFELDGKQTTVADYFRSRKYNLMYPNLLCLHVGPPLKN 471

Dper\Ago2c CLASAPRVFRVNGLTKFPASSLKFELDGKQTTVADYFRSRKYNLMYPNLLCLHVGPPLKN 456

************************************************************

Dpse\Ago2c IYLPIELCRIEDGQALNRKDGANQVAAMIKYAATSTNERKAKIIHLLEYFKHNLDPTISH 531

Dper\Ago2c IYLPIELCRIEDGQALNRKDGANQVAAMIKYAATSTNERKAKIIHLLEYFKHNLDPTISH 516

************************************************************

Dpse\Ago2c FGIRLENDFIVVHTRTLNAPQVEYKNNNLASVRNGSWRMDRMQFFEPKPKPHKWAILHGK 591

Dper\Ago2c FGIRLENDFIVVHTRTLNAPQVEYKNNNLASVRNGSWRMDRMQFFEPKPKPHKWAILHGK 576

************************************************************

Dpse\Ago2c INYMSVVDFQGMIIQQSRTVNVCLNQKADIRNYRDERELDSHFQDFKKNQFDLVFVIIPN 651

Dper\Ago2c INYMSVVDFQGMIIQQSRTVNVCLNEKADIRNYRDERELDSHFQDFKKNQFDLVFVIIPN 636

*************************:**********************************

Dpse\Ago2c SGPFYDVVKQKAELQHGILTQCIKEITVLRKCNLQCIGNVLLKVNSKLNGINHKLKDDPR 711

Dper\Ago2c SGPFYDVVKQKAELQHGILTQCIKEITVLRKCNLQCIGNVLLKVNSKLNGINHKLKDDPR 696

************************************************************

Dpse\Ago2c FLLKNAMFLGADVTHPSPDQREIPSVVGVAASHDPFGASYNMQYRLQRSALEEIEDMESI 771

Dper\Ago2c FLLKNAMFLGADVTHPSPDQREIPSVVGVAASHDPFGASYNMQYRLQRSALEEIEDMESI 756

************************************************************

Dpse\Ago2c TLEHFRVYHQFRKSYPEHIVYYRDGVSDGQFPKIKNEELRGISAACSKLRINPKICCVIV 831

Dper\Ago2c TLEHLRVYHQFRKSYPEHIVYYRDGVSDGQFPKIKNEELRGISAACSKMRINPKICCVIV 816

****:*******************************************:***********

Dpse\Ago2c VKRHHTRFFPNGAPSQYNKFNNVDPGTVVDRTIVHPNEMQFFMVSHQSIQGTAKPTRYNV 891

Dper\Ago2c VKRHHTRFFPNGAPSQYNKFNNVDPGTVVDRTIVHPNEMQFFMVSHQSIQGTAKPTRYNV 876

************************************************************

Dpse\Ago2c IENTGNLDIDLLQQLTYNLCHMFPRCNRAVSYPAPAYLAHLAAARGRVYLTGCTKFRTPK 951

Dper\Ago2c IENTGNLDIDLLQQLTYNLCHMFPRCNRAVSYPAPAYLAHLAAARGRVYLTGCTKFRTPK 936

************************************************************

Dpse\Ago2c EEYAKRLIVPEFMKTNPMYFV 972

Dper\Ago2c EEYAKRLIVPEFMKTNPMYFV 957

*********************
