# Supplementary material for: Natural Variation of the Amino-Terminal Glutamine-Rich Domain in Drosophila Argonaute2 Is Not Associated with Developmental Defects
Source: PLoS One. 2010 Dec 17;5(12):e15264. doi: 10.1371/journal.pone.0015264 (PMC3002974; doi:10.1371/journal.pone.0015264)
Supplement: Table S1 — Evidence for expression of Ago2 NTDs in various insect species. For various insect Ago2s, the genomic regions corresponding to the NTD are indeed transcribed. Evidence includes cDNA analysis of Ago2 specifically, EST data from high-throughput sequencing efforts, and RNAseq data. If the transcribed region encompasses the entire NTD, this is indicated as “full” in the table. (DOC) [file pone.0015264.s006.doc]

**Table S1: Evidence for expression of Ago2 NTDs in various insect species**

For various insect Ago2s, the genomic regions corresponding to the NTD are indeed transcribed. Evidence includes cDNA analysis of Ago2 specifically, EST data from high-throughput sequencing efforts, and RNAseq data. If the transcribed region encompasses the entire NTD, this is indicated as “full” in the table.

| **species** | **Name of protein** | **Type of data** | **Partial or full NTD?** | **GenBank entry** | **references** |
| --- | --- | --- | --- | --- | --- |
| *D. melanogaster* | Dmel\Ago2 | cDNA | full | BT003546, BT099682 | Hammond et al., (2001); Meyer et al., (2006); this study |
| *D. simulans* | Dsim\Ago2 | cDNA | full |  | This study |
| *D. sechellia* | Dsec\Ago2 | cDNA | full |  | This study |
| *D. erecta* | Dere\Ago2 | EST | partial | EB541473 |  |
| *D. pseudoobscura* | Dpse\Ago2c | RNAseq | partial |  | Malon and Oliver (2010) |
| *D. pseudoobscura* | Dpse\Ago2d | RNAseq | partial |  | Malon and Oliver (2010) |
| *D. willistoni* | Dwil\Ago2a | EST | partial | EB457635 |  |
| *D. mojavensis* | Dmoj\Ago2 | EST | partial | EB601488 |  |
| *N. vitripennis* | Nvit\Ago2 | EST | full | GE432210 and others |  |
| *A. pisum* | Apis\Ago2 | EST | partial | FF292656 and others |  |
| *Anopheles gambiae* |  | EST | partial | BM625231 | Meyer et al. (2006) |
| *Diabrotica virgifera* |  | EST | partial | EW777225 |  |

Malone, J.H., Oliver, B. (2010.3.15). PREVIEW: mRNA-Seq of head tissue from *Drosophila* pseudoobscura.; Personal communication to FlyBase; FlyBase ID FBrf0210196.
